# Lipid functions in skin: Differential effects of n-3 polyunsaturated fatty acids on cutaneous ceramides, in a human skin organ culture model^☆^

**DOI:** 10.1016/j.bbamem.2017.03.016

**Published:** 2017-09

**Authors:** Alexandra C. Kendall, Magdalena Kiezel-Tsugunova, Luke C. Brownbridge, John L. Harwood, Anna Nicolaou

**Affiliations:** a Laboratory for Lipidomics and Lipid Biology, Division of Pharmacy and Optometry, School of Health Sciences, Faculty of Biology, Medicine and Health, University of Manchester, Manchester Academic Health Science Centre, Manchester M13 9PL, UK; b School of Biosciences, Cardiff University, Cardiff CF10 3AX, UK

**Keywords:** AA, arachidonic acid, C1P, ceramide-1-phosphate, CB, cannabinoid receptor, CER[*ADS*], alpha-hydroxy fatty acid/dihydrosphingosine base ceramide, CER[*AH*], alpha-hydroxy fatty acid/6-hydroxy-sphingosine base ceramide, CER[*AP*], alpha-hydroxy fatty acid/phytosphingosine base ceramide, CER[*AS*], alpha-hydroxy fatty acid/sphingosine base ceramide, CER[EODS], ester-linked omega-hydroxy fatty acid/dihydrosphingosine base ceramide, CER[*EOH*], ester-linked omega-hydroxy fatty acid/6-hydroxy-sphingosine base ceramide, CER[*EOP*], ester-linked omega-hydroxy fatty acid/phytosphingosine base ceramide, CER[*EOS*], ester-linked omega-hydroxy fatty acid/sphingosine base ceramide, CER[*NDS*], non-hydroxy fatty acid/dihydrosphingosine base ceramide, CER[*NH*], non-hydroxy fatty acid/6-hydroxy-sphingosine base ceramide, CER[*NP*], non-hydroxy fatty acid/phytosphingosine base ceramide, CER[*NS*], non-hydroxy fatty acid/sphingosine base ceramide, DHA, docosahexaenoic acid, DS, dihydrosphingosine, DS1P, dihydrosphingosine-1-phosphate, EPA, eicosapentaenoic acid, ESI, electrospray ionisation, H, 6-hydroxysphingosine, LA, linoleic acid, MS, mass spectrometry, MS/MS, tandem mass spectrometry, n-3PUFA, omega-3 polyunsaturated fatty acid, P, phytosphingosine, PUFA, polyunsaturated fatty acid(s), S, sphingosine, S1P, sphingosine-1-phosphate, UPLC, ultraperformance liquid chromatography, Skin, Ceramides, Omega-3 fatty acids, Mass spectrometry, Lipidomics

## Abstract

Ceramides are important for skin health, with a multitude of species found in both dermis and epidermis. The epidermis contains linoleic acid-**E**ster-linked **O**mega-hydroxylated ceramides of 6-**H**ydroxy-sphingosine, **S**phingosine and **P**hytosphingosine bases (CER[*EOH*], CER[*EOS*] and CER[*EOP*], respectively), that are crucial for the formation of the epidermal barrier, conferring protection from environmental factors and preventing trans-epidermal water loss. Furthermore, a large number of ceramides, derivatives of the same sphingoid bases and various fatty acids, are produced by dermal and epidermal cells and perform signalling roles in cell functions ranging from differentiation to apoptosis.

Supplementation with the n-3 polyunsaturated fatty acids (PUFA) eicosapentaenoic acid (EPA) and docosahexaenoic acid (DHA) have shown promise as therapeutic agents in a number of inflammatory skin conditions, altering the lipid profile of the skin and production of bioactive lipids such as the eicosanoids, docosanoids and endocannabinoids. In this study we wished to investigate whether EPA and DHA could also affect the ceramide profile in epidermis and dermis, and, in this way, contribute to formation of a robust lipid barrier and ceramide-mediated regulation of skin functions.

*Ex vivo* skin explants were cultured for 6 days, and supplemented with EPA or DHA (50 μM). Liquid chromatography coupled to tandem mass spectrometry with electrospray ionisation was used to assess the prevalence of 321 individual ceramide species, and a number of sphingoid bases, phosphorylated sphingoid bases, and phosphorylated ceramides, within the dermis and epidermis.

EPA augmented dermal production of members of the ceramide families containing **N**on-hydroxy fatty acids and **S**phingosine or **D**ihydrosphingosine bases (CER[*NS*] and CER[*NDS*], respectively), while epidermal CER[*EOH*], CER[*EOS*] and CER[*EOP*] ceramides were not affected. DHA did not significantly affect ceramide production. Ceramide-1-phosphate levels in the epidermis, but not the dermis, increased in response to EPA, but not DHA.

This *ex vivo* study shows that dietary supplementation with EPA has the potential to alter the ceramide profile of the skin, and this may contribute to its anti-inflammatory profile. This has implications for formation of the epidermal lipid barrier, and signalling pathways within the skin mediated by ceramides and other sphingolipid species. This article is part of a Special Issue entitled: Membrane Lipid Therapy: Drugs Targeting Biomembranes edited by Pablo V. Escribá.

## Highlights

• Omega-3 fatty acid supplementation alters *ex vivo* skin ceramide profiles
• Eicosapentaenoic acid (EPA) increases dermal ceramides with non-hydroxy fatty acids (CER[*NS*] and CER[*NDS*])
• EPA increases ceramide-1-phosphate (C1P) in the epidermis but not dermis
• Long-chain linoleic-acid-containing ceramides were unaltered by EPA or docosahexaenoic acid (DHA)

## Graphical abstract

**Figure:**
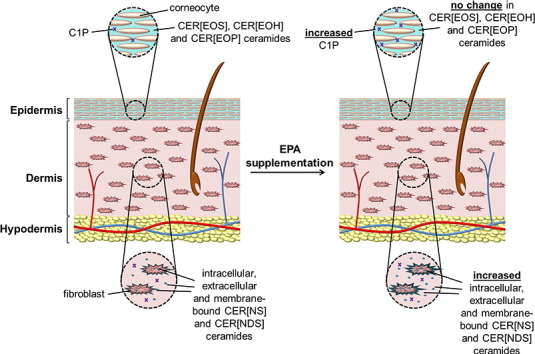

## 1 Introduction

### 1.1 Skin physiology is supported by distinct lipid metabolism

The skin is the largest organ of the body and functions as a barrier preventing water loss and entry of harmful compounds or organisms from the environment, as well as offering protection against solar radiation [1]. It also allows sensations of pain, temperature and touch, contributes to thermoregulation, and mediates inflammatory and immune responses. The skin is characterised by active lipid metabolism, with a distinct profile of lipids, and is the source of an array of bioactive lipid mediators [2].

Skin is composed of three main layers, the dermis, the epidermis and the hypodermis, and because of its protective function, is continuously dynamic. Epidermal keratinocytes differentiate as they migrate upwards, eventually becoming corneocytes. These protein-enriched corneocytes are embedded in a lipid-rich matrix comprising a large number of ceramide species, cholesterol and free fatty acids, to form the *stratum corneum* [3], [4]. Also found in the epidermis are the antigen-presenting Langerhans cells and pigment-producing melanocytes [2]. The dermis contains a number of important structures (small blood vessels and nerves, hair follicles and sweat glands) together with dermal fibroblasts and immune cells, all enclosed within collagen and elastic fibres [2], [5]. The subcutaneous tissue (hypodermis) that forms the lowest of the skin layers contains blood vessels and adipocytes sufficient in number to form adipose tissue. Although adipose tissue has its major role as a lipid storage site, it is also a source of fatty acid-derived lipid mediators with signalling properties [6]. Sebum-derived lipids are found on the surface of the skin; this complex mixture of triacylglycerols, diacylglycerols, non-esterified fatty acids, wax esters, squalene and cholesterol esters offer photoprotection and exert anti-microbial activities, although the entire spectrum of its role is not yet fully understood [7], [8].

Importantly, it has been shown that epidermal keratinocytes have very low desaturase activity, resulting in poor ability to form long chain polyunsaturated fatty acids (PUFA), including arachidonic acid (AA; 20:4n-6) [9]. This highlights the importance of systemic long chain PUFA supplementation for skin health, and the role of dermal-epidermal cross talk for the efficient structure and function of the epidermis. Indeed, the essential fatty acid linoleic acid (LA; C18:2n-6) is of particular significance to skin health, as it contributes to the formation of ceramides essential for the structure of the epidermal barrier [3], and the absence of LA-containing ceramides in the stratum corneum results in barrier permeability problems [10], [11], [12].

As well as contributing to the structural integrity of the skin, PUFA such as LA, AA, eicosapentaenoic acid (EPA; C20:5n-3) and docosahexaenoic acid (DHA; C22:6n-3) are metabolized to octadecanoids, eicosanoids, docosanoids, endocannabinoids and related bioactive lipid species, known to mediate inflammatory and immune reactions in many tissues, including skin [13], [14], [15], [16], [17].

### 1.2 Sphingolipids perform unique roles within the skin

Comprising both signalling and structural lipids, the cutaneous sphingolipids are crucial for skin health. Sphingoid bases, ceramides and their phosphorylated derivatives have been analysed, and hundreds identified in skin [18], [19], [20], [21], [22], [23], [24]. Whilst the functions of the signalling sphingolipid species in other mammalian tissues continue to be elucidated [25], knowledge of their function in skin is rather sparse [26]. However, the role of structural ceramides in forming the skin barrier is better understood.

As discussed, the barrier function of the skin depends on the presence of a specific mixture of lipids within the intercellular spaces of the *stratum corneum* [27]. Towards the end of the keratinocyte differentiation process, synthesised lipids that have accumulated in lamellar granules are discharged and released by the action of enzymes co-secreted from the lamellar bodies (including phospholipases, sphingomyelinase and β-glucocerebrosidase) [4], [28]. These are the main source of the barrier lipids, and diseases with changes in activity of such enzymes give rise to abnormal barrier function (e.g [29].). Barrier lipids represent about 10% of the mass of the *stratum corneum*, and consist of about 50% ceramides, 25% cholesterol and 15% non-esterified fatty acids [30], [31], [32]. The composition and organisation of these lipids has been discussed recently by van Smeden et al. [33].

**Fig. 1:**
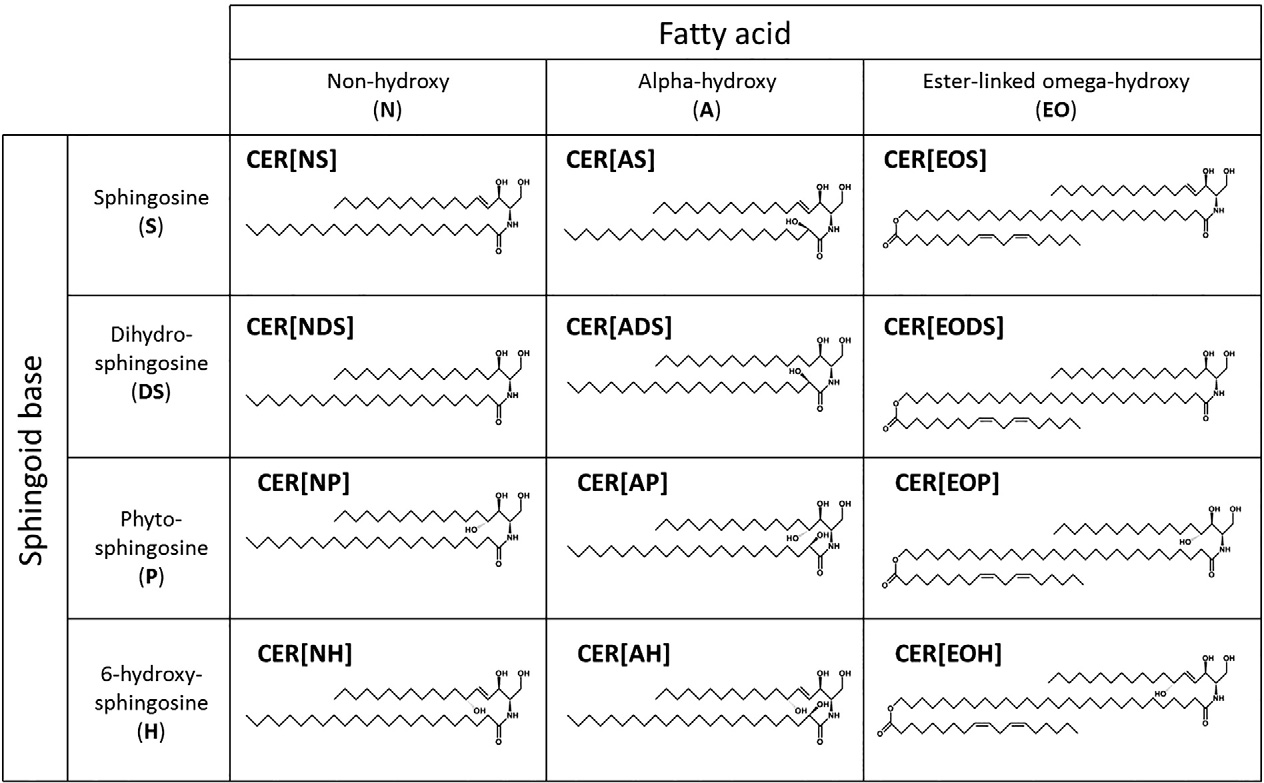
The ceramides are a complex family of sphingoid-based lipids. Nomenclature of the different ceramide families is based on the sphingoid base (sphingosine, S; dihydrosphingosine (also known as sphinganine), DS; phytosphingosine, P; 6-hydroxysphingosine; H) and fatty acid (non-hydroxy, H; alpha-hydroxy, A; ester-linked omega-hydroxy, EO). Different combinations of sphingoid base and fatty acid generate the different ceramide families, for example a non-hydroxy fatty acid (N) amide-linked to a sphingosine (S) base creates a CER[*NS*] ceramide. Individual ceramide species are further identified by carbon chain lengths of both base and fatty acid. For example, an NS ceramide with a 30-carbon non-hydroxy fatty acid and a 20-carbon sphingosine base is denoted as N(30)S(20). The above ceramide families have all been identified in human skin [21].

The ceramides are of particular importance, and display extreme complexity due to a multitude of substructures comprising different sphingoid bases and fatty acids (see Fig. 1 for ceramide families, including structures and an explanation of the nomenclature) including some very long chain N-acyl fatty acids. Notably, there is a sub-group of ceramides that contain LA esterified to the omega carbon of omega-hydroxy fatty acids (CER[*EOS*], CER[*EODS*], CER[*EOP*] and CER[*EOH*]; Fig. 1). These are found solely in the epidermis and help to form the multilayers of membrane sheets, crucial for skin barrier function [20].

In essential fatty acid deficiency, there is an abnormal appearance of the extracellular lipid membrane that is commensurate with impaired barrier function, which reflects failure to synthesise these ceramide families [34], [35]. The important role of LA in barrier function is discussed by Elias et al. [3]. Additionally, changes in individual ceramide proportions have a noticeable effect on barrier conformation and, hence, its properties [36].

### 1.3 n-3PUFA supplementation and skin sphingolipids

The importance of PUFA in skin health is clear, especially the potential benefits of n-3PUFA supplementation, which has been shown in several studies to ameliorate some inflammatory skin conditions [15], [37], [38], [39], [40]. Additionally, the crucial role of ceramides in maintaining a healthy epidermal barrier has been thoroughly examined.

*Ex vivo* skin organ culture has long been used as a model system for the assessment of skin physiology and is a useful system for the study of skin lipids [41], [42]. Organ culture systems similar to the one used in this study have shown that skin can be kept viable for up to 4 weeks [43], [44], [45], [46] and maintain physiological processes including irritant-induced Langerhans cell migration [47], and release of inflammatory mediators including eicosanoids, endocannabinoids, interleukin-1 and histamine [16], [48], [49].

Previously, our group has explored the PUFA-derived bioactive lipids present in human epidermis and dermis, and reported the presence of a range of prostanoids, hydroxy fatty acids, endocannabinoids and N-acyl ethanolamides [16]. Using *ex vivo* skin as a means of manipulating the cutaneous fatty acid profile under controlled conditions, we showed that provision of EPA or DHA through the culture media perturbed some existing lipid mediators and also gave rise to anti-inflammatory lipid species in both the dermis and the epidermis [16]. In the present study, we wished to expand this analysis and examine the effect of n-3PUFA supplementation on the ceramide profile of the skin, aiming to explore whether n-3PUFA have the potential to affect the formation and integrity of the barrier through changes in the main lipid component of the epidermis - structural ceramides - as well as signalling members of the sphingolipid family found in both the epidermis and dermis.

## 2 Materials and methods

### 2.1 Materials

Cell culture reagents, fatty acids and LC/MS grade solvents were purchased from Sigma Aldrich (Poole, UK). Calcium chloride was purchased from Promocell (Heidelberg, Germany). Internal standard cocktail (Ceramide/Sphingoid Internal Standard Mixture I) was purchased from Avanti Polar Lipids, Alabaster, Alabama, USA).

### 2.2 Skin samples

Skin was obtained from the Ethical Tissue biobank (University of Bradford, Bradford, UK) with full ethical approval (Leeds East Research Ethics Committee reference 07/H1306/98+5). Skin was obtained with informed consent from four healthy female donors (33–47 years; white Caucasian), who were undergoing elective abdominoplasty surgery. Samples were delivered to the biobank within 1 h of surgery, and then to the laboratory within 12 h of this (refrigerated if overnight storage was necessary).

### 2.3 *Ex vivo* skin organ culture

Skin tissue was washed in PBS containing 100 U/ml penicillin, 100 μg/ml streptomycin and 2.5 mg/ml amphotericin B, and the adipose layer was removed. Punch biopsies (6 mm diameter) were cut from the tissue, and cultured in 24-well plates with 500 μl DMEM (supplemented with 100 U/ml penicillin, 100 μg/ml streptomycin and 1.4 mM Ca^2 +^) as previously reported [16]. The Ca^2 +^ (1.4 mM) was added in the form of CaCl_2_, directly to the media. Exogenous calcium has been shown to maintain keratinocyte and fibroblast survival in full-thickness human skin [41], [50], [51]. Fatty acid supplementation was performed by dissolving EPA or DHA in DMSO and adding this (2 μl per 10 ml medium, or an equal volume of vehicle (DMSO)) directly to the media to a final concentration of 50 μM. Fatty acid treatments were prepared and replaced daily, for a total of 6 days of culture. A concentration of 50 μM EPA/DHA was chosen as we have found this to be sufficient to alter cellular fatty acid levels without a risk of toxicity in epidermal keratinocyte and dermal fibroblast monolayers (unpublished data). Lack of toxicity at this dose has also been published previously [52], [53], [54]. In the present study, the viability of skin tissue and lack of toxicity of the fatty acid supplement were assessed *via* analysis of lactate dehydrogenase (LDH) release, using media removed from the culture wells each day (LDH Cytotoxicity Assay Kit; Cayman Chemical, Ann Arbor, USA) (Supplementary data S3). EPA/DHA uptake was confirmed by GC-FID analysis (1 punch biopsy, 120–250 mg), as previously described [55] (Supplementary data S3). After 6 days in culture, skin biopsies were washed in PBS, snap-frozen and stored at − 80 °C for lipidomic analysis.

### 2.4 Analysis of lipids by UPLC/ESI-MS/MS

Skin (1 punch biopsy per time point, 120–250 mg) was divided into dermis and epidermis by scalpel at × 40 magnification, and lipids were extracted as previously described [16], [17]. Although a scalpel does not produce a completely clean separation of the dermis and epidermis, and some dermal contamination of the epidermis remains, this method prevents any degradation of the lipids that occurs during other separation techniques that depend on incubation in salts, enzymes or extreme temperatures [56], [57], and care was taken not to allow any epidermal contamination of the dermis. We have previously shown this technique to reveal clear differences in the lipid profiles of the two skin compartments [16]. Dermal tissue weighed 100–200 mg, while epidermal sections weighed 20–55 mg. Tissue was homogenised in ice-cold isopropanol:water:ethyl acetate (30:10:60; *v*/v/v; 4 ml per sample) using a blade homogeniser (X 10/25 drive with 10 mm diameter shaft, set at a speed of 11 kHz; Ystral, Ballrechten-Dottingen, Germany) for 3 × 3 s pulses, on ice. Internal standards were added (a cocktail containing 50 pmol each of C17 S, C17 DS, C17 S1P, C17 DS1P, C12 C1P and C25 Cer) and the samples were incubated on ice for 90 min. Samples were centrifuged to remove protein precipitate and the supernatant was dried down under nitrogen, before lipid extracts were reconstituted in methanol with 0.1% (*v*/v) formic acid.

Lipidomic analysis was performed using ultraperformance liquid chromatography (Acquity UPLC; Water, Wilmslow, UK) coupled to a triple quadrupole mass spectrometer with electrospray ionisation (Xevo TQ-S; Water, Wilmslow, UK). Analytes were separated using a C8 column (Acquity UPLC BEH, 1.7 μm, 2.1 × 100 mm; Waters, Wilmslow, UK) and a gradient of solvent A (water with 0.1% formic acid) and solvent B (methanol with 0.1% formic acid) at a flow rate of 0.3 ml/min as follows: 60% B (0–6 min), 60–96% B (6–9 min), 96–100% B (9–20 min), 100% B (20–30 min), 100–60% B (30–32 min), 60% B (32–40 min). Analytes were fragmented using argon as a collision gas and monitored in the positive ion mode by multiple reaction monitoring (MRM) (parameters given in Supplementary data S1). Relative quantification of analytes was performed using the class-specific internal standards described above.

### 2.5 Protein content

Following tissue homogenisation and extraction of the lipids from the biopsies, the protein precipitate was collected and the protein content measured using Bio—Rad Protein Assay II [58] (Bio—Rad, Hemel Hempstead, UK).

### 2.6 Statistical analysis

Friedman tests followed by Dunn's multiple comparisons tests for differences in ceramide and lipid mediator expression were performed using GraphPad Prism version 7.00. *P* < 0.05 was considered significant.

## 3 Results

### 3.1 Ceramide species from 11 families were identified in skin explants

**Fig. 2:**
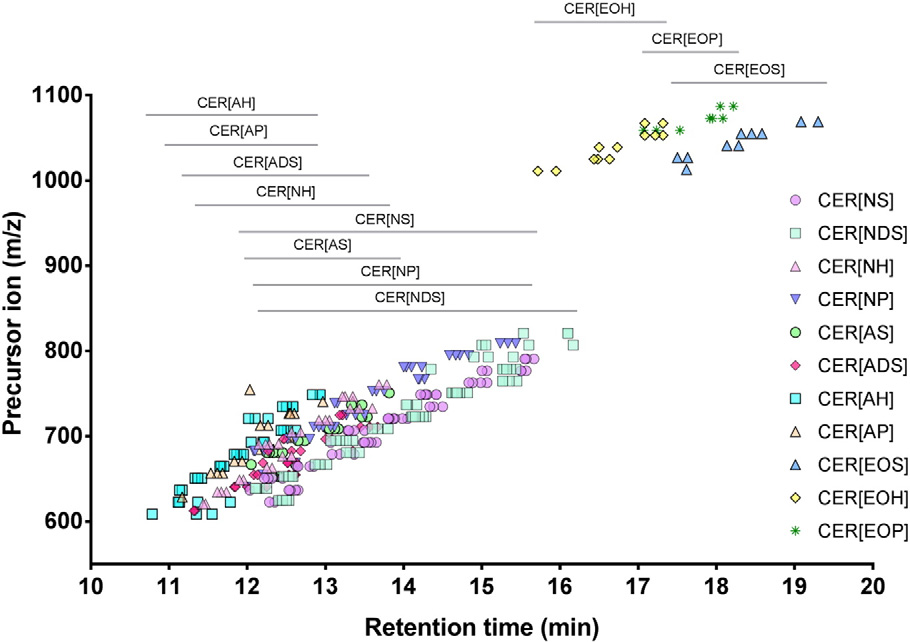
Ceramide species from 11 different families were identified in human dermis and epidermis. Analytes were separated by reverse-phase ultraperformance liquid chromatography using a C8 column, and identified using a triple-quadrupole mass spectrometer with electrospray ionisation. Identification was performed by multiple reaction monitoring and retention time.

**Fig. 3:**
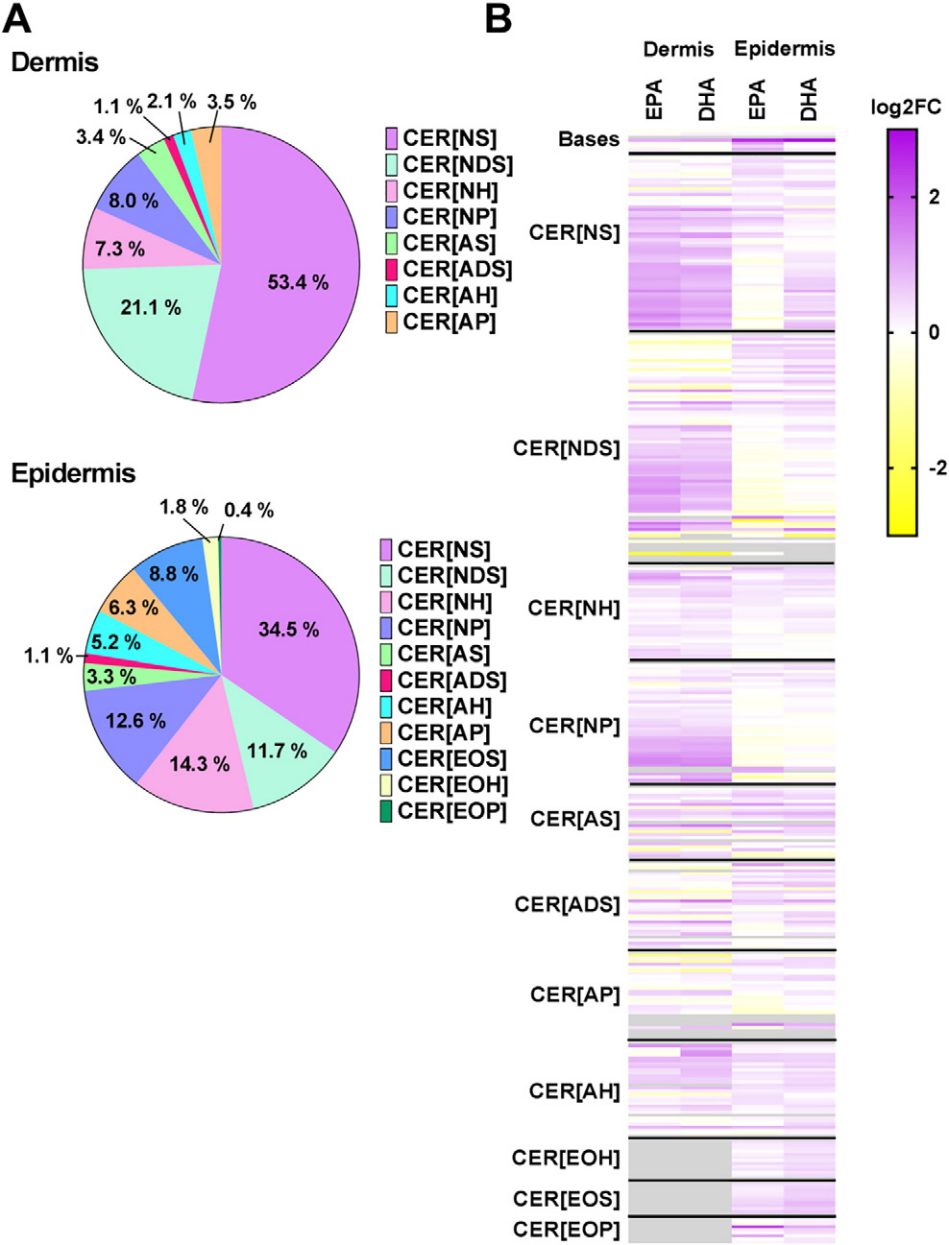
Matched skin samples from four donors were cultured, with or without EPA or DHA supplementation (50 μM) for 6 days. Skin was separated into dermis and epidermis and the ceramide expression was quantified by UPLC/ESI-MS/MS. The proportions of ceramide families were different in the dermis and epidermis (**A**). Data shown are from control samples, as % of total ceramides detected. The skin showed some changes in sphingolipid expression following supplementation with EPA or DHA (**B**). Data are shown as the mean log2 fold change compared with control samples of four donors. Grey boxes represent missing data (no expression in control or treated samples).

Using UPLC/ESI-MS/MS we measured the prevalence of 321 ceramide species from 11 different families in the dermis and epidermis of *ex vivo* skin explants (Fig. 2). These comprised 57 CER[*NS*], 75 CER[*NDS*], 30 CER[*NH*], 39 CER[*NP*], 23 CER[*AS*], 28 CER [ADS], 19 CER[*AH*], 20 CER[*AP*], 10 CER[*EOS*], 8 CER[*EOP*] and 12 CER[*EOH*] ceramide species (Supplementary data S2). Of these, only 16 dermal species (6 CER[*NDS*], 2 CER[*AS*], 1 CER[*ADS*] and 7 CER[*AH*]) and 15 epidermal species (6 CER[*NDS*], 2 CER[*AS*], 1 CER[*ADS*] and 6 CER[*AH*]) were below the limit of detection. As expected, the CER[*EOS*], CER[*EOH*] and CER[*EOP*] families were only found in the epidermis, while all other families were found in both the dermis and epidermis, although the proportions of ceramide families did vary between the two skin compartments (Fig. 3A).

Following supplementation of the *ex vivo* skin explants with EPA or DHA (50 μM for 6 days), the ceramides showed some changes in expression, which were more apparent in the dermis than the epidermis (Fig. 3B). Although there was no statistically significant difference in the total ceramide pool, or the totals of the 11 ceramide families (data not shown), there were differences in individual ceramide species.

### 3.2 CER[*NS*] and CER[*NDS*] ceramides increase in the dermis in response to EPA supplementation

**Fig. 4:**
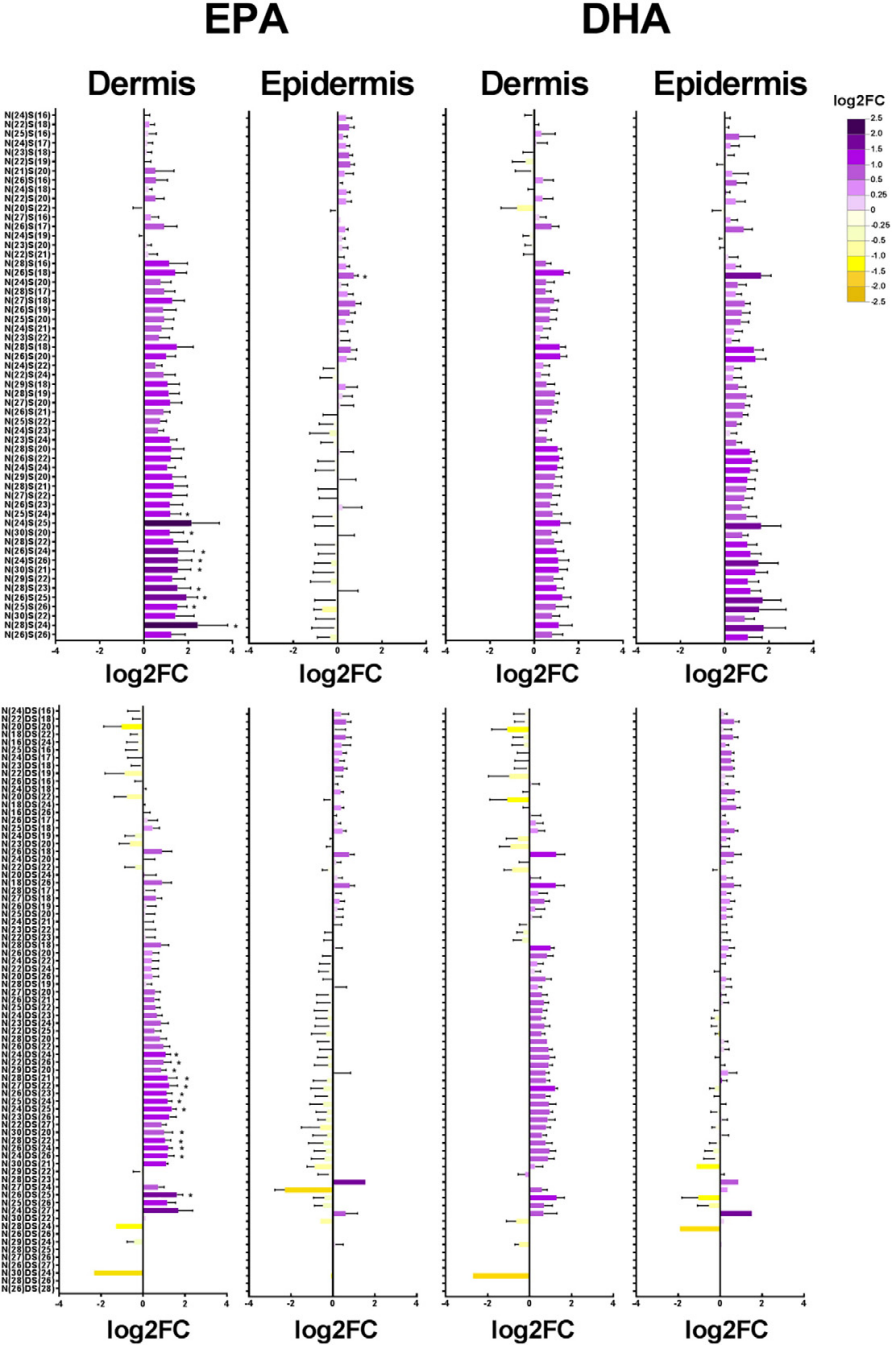
CER[*NS*] and CER[*NDS*] ceramide species in the dermis and epidermis following supplementation with EPA and DHA. Data are shown as log2 fold changes from control, mean ± SEM of four skin donors. *P < 0.05 *vs* control.

Following EPA supplementation, expression of nine CER[*NS*] and 13 CER[*NDS*] species was found statistically significantly increased in the dermis (Fig. 4). These CER[*NS*] and CER[*NDS*] species were N(25)S(24), N(30)S(20), N(26)S(24), N(24)S(26), N(30)S(21), N(28)S(23), N(26)S(25), N(25)S(26) and N(28)S(24), and N(24)DS(24), N(22)DS(26), N(29)DS(20), N(28)DS(21), N(27)DS(22), N(26)DS(23), N(25)DS(24), N(24)DS(25), N(30)DS(20), N(28)DS(22), N(26)DS(24), N(24)DS(26), and N(26)DS(25), respectively. These changes were not observed in the epidermis, where EPA had no significant effect on CER[*NS*] or CER[*NDS*] ceramide expression. Although there was a trend for increased CER[*NS*] in the dermis and epidermis, and CER[*NDS*] expression in the dermis, following DHA supplementation, this did not reach statistical significance. There was considerable variation between skin donors in their response to EPA or DHA supplementation.

### 3.3 Levels of other ceramide families did not change in the dermis or epidermis in response to n-3PUFA supplementation

The LA-containing CER[*EOS*], CER[*EOH*] and CER[*EOP*] families were present only in the epidermis (Fig. 3), and there was no overall trend in response to EPA or DHA supplementation, and no significant change in any of the individual species measured in our assay.

The other ceramide families (CER[*NH*], CER[*NP*], CER[*AS*], CER[*ADS*], CER[*AH*], and CER[*AP*]) showed no significant change in expression following supplementation with EPA or DHA, in the dermis or epidermis. Again there was variation between skin donors but no consistent increase or decrease following fatty acid supplementation (Fig. 3).

### 3.4 Sphingoid bases and phosphorylated species

**Fig. 5:**
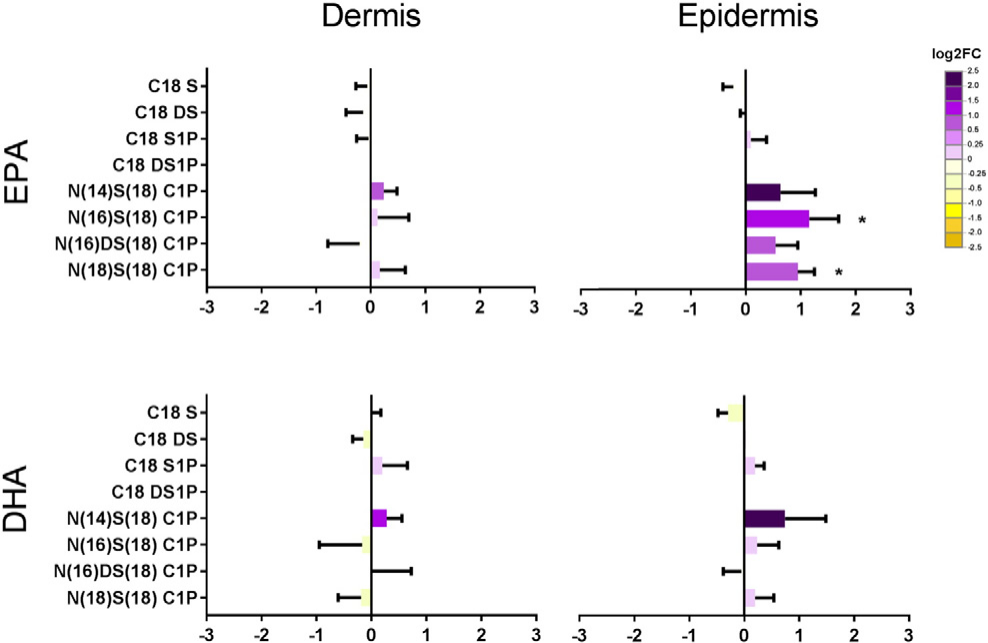
Sphingoid bases, S1Ps and C1Ps in the dermis and epidermis following supplementation with EPA and DHA. Data are shown as log2 fold changes from control, mean ± SEM. *P < 0.05 *vs* control.

The 18 carbon sphingosine (S) and dihydro-sphingosine (DS) bases and sphingosine-1-phosphate (S1P) were present in the dermis and epidermis, but showed no change in expression following EPA or DHA supplementation (Fig. 5). DS1P was present only in concentrations around the limit of detection.

### 3.5 Phosphorylated ceramide species

We measured four different ceramide-1-phosphate (C1P) species in the dermis and epidermis in control skin and following supplementation with EPA or DHA (Fig. 5). Two of these species, N(16)S(18)C1P and N(18)S(18)C1P were the most abundant, and showed a clear statistically significant upregulation in EPA-treated epidermis compared with control or DHA-treated epidermis (Fig. 5). There was no difference in C1P levels in the dermis following n-3 supplementation.

## 4 Discussion

The importance of dietary fatty acids for skin health has long been known [59], [60]. N-3PUFA supplementation has been shown to be beneficial for skin health [2], [15], [37], [40], and since ceramides are crucial in normal skin function, we were interested in whether manipulation of the skin's fatty acid profile following n-3PUFA treatment would have an impact on ceramide production in the dermis and epidermis. We have profiled 321 ceramide species in human dermis and epidermis using a semi-quantitative UPLC/ESI-MS/MS method [16]. Identification of individual ceramide species by fragmentation pattern and retention time has allowed us to perform relative quantification using internal standards [18], [19], [21]. This analysis includes CER[*NS*], CER[*NDS*], CER[*NH*], CER[*NP*], CER[*AS*], CER[*ADS*], CER[*AH*] and CER[*AP*] species that may have roles in inter-cellular signalling, as well as CER[*EOS*], CER[*EOH*] and CER[*EOP*] species that are crucial for successful epidermal barrier formation [4], [26]. In addition, we have examined some of the related bioactive lipid mediators, including S1P and C1P species known to have signalling roles within the skin [26], [61], [62].

Several species of CER[*NS*] and CER[*NDS*] showed increased expression in the dermis, but not epidermis, in response to EPA. The ceramide species that increased in response to EPA were among the least abundant of the CER[*NS*] and CER[*NDS*] families (Supplementary data S2). Analysis of ceramide family totals showed no significant differences following EPA supplementation (data not shown), and the increases were specific to these low-abundance species. This indicates the response is not an artefactual finding due to low basal amounts of these species. Although a similar trend was observed with DHA supplementation, this did not reach statistical significance for any ceramide species. This was due to variation in the responses of skin from different donors, and is supported by previous reports on inter-individual variation in the uptake and response to n-3PUFA [63]. The n-3PUFA-induced increases were not seen with any of the other ceramide families, and overall, we observed that the skin's ceramide profile was largely unaffected by provision of EPA or DHA. To date, we have been unable to find any reports on the effect of EPA and DHA on ceramides throughout the dermis and epidermis, and this absence of global ceramide changes may highlight the complexity of ceramide metabolism in the skin.

Previous studies on the influence of n-3PUFA on ceramides in tissues other than skin show tissue-dependent responses. In murine muscle cells, EPA or DHA added independently at a concentration of 50 μM have both been shown to impair a palmitate-induced increase in CER[*NS*] [64], whilst *in vivo* dietary fish oil supplementation (which contains EPA and DHA together) in mice can inhibit a high-fat-diet-induced CER[*NS*] increase in skeletal muscle [65]. It has also been shown in humans that adopting a Nordic diet (which includes high fish consumption), leads to reduction in some plasma CER[*NS*] ceramides, although the originating tissue of these species is unknown [66]. This variation between tissue responses is evident within the same animal, as mice on fish oil- or krill oil-supplemented diets had decreased CER[*NS*] and CER[*NDS*] ceramides in the liver, and increased CER[*NS*] and CER[*NDS*] ceramides in the brain [67]. Increased ceramide production has also been seen in breast cancer cells following EPA or DHA supplementation *in vitro*, or fish oil supplementation *in vivo*, through increased neutral sphingomyelinase activity [68].

Given the signalling roles of CER[*NS*] and CER[*NDS*] species, their reported increase in the dermis in response to EPA supplementation may contribute to regulation of fibroblast functions, such as cell differentiation and apoptosis [26]. However, since the identification of individual ceramide species is a relatively recent development in lipidomics, there is little evidence for the roles of specific ceramides, and we have been unable to find any previous studies on the individual CER[*NS*] and CER[*NDS*] species we found to be altered by EPA. As the field advances, we expect studies to be performed using individual ceramide species, which may elucidate their roles.

**Fig. 6:**
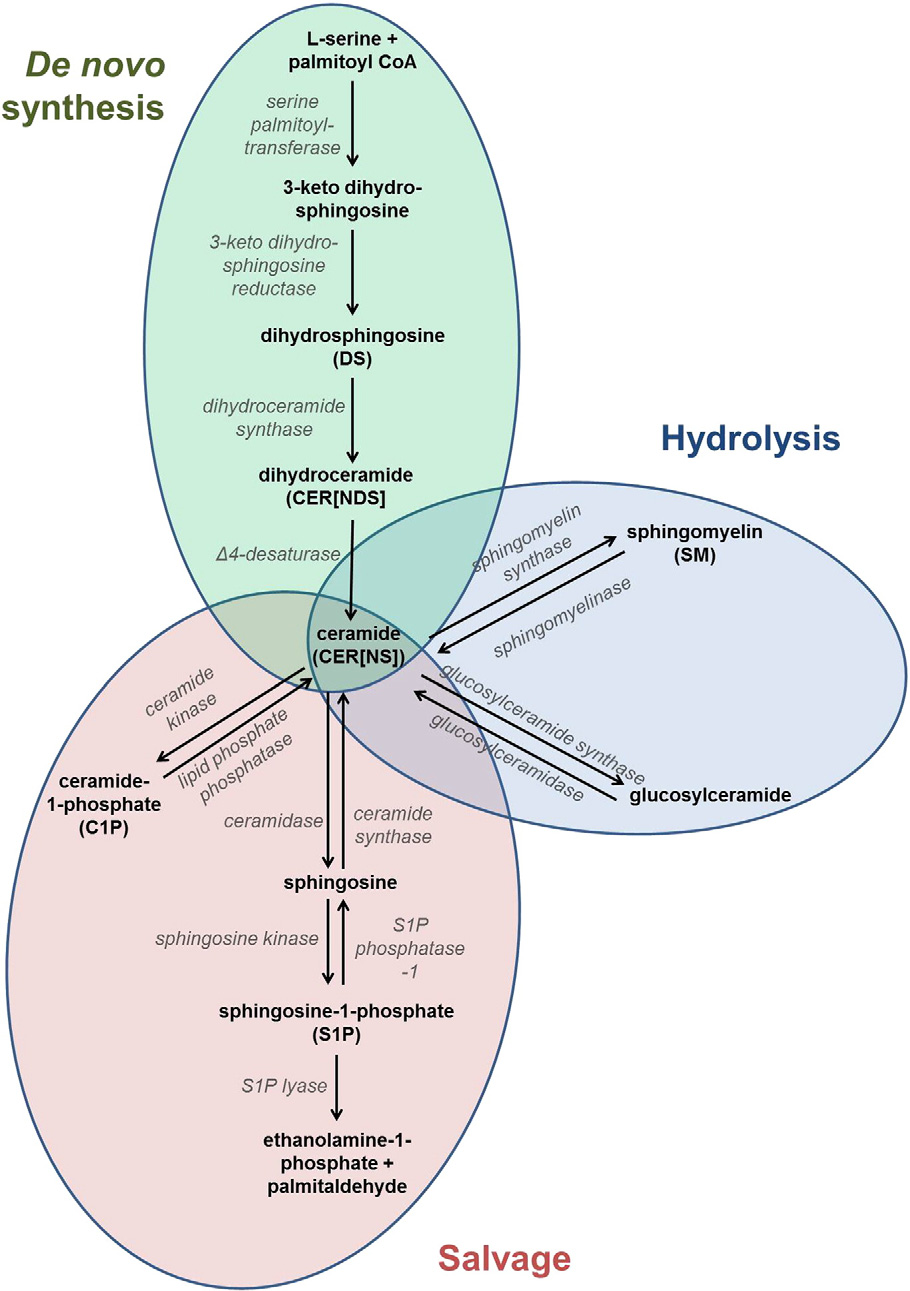
The sphingolipid cycle allows ceramides to be metabolized through various pathways, including *de novo* synthesis, presence in sphingomyelins or glucosylceramides, or production of phosphorylated signalling species. There are multiple potential targets through which n-3PUFA could alter ceramide levels.

The involvement of sphingomyelinase is a possible explanation for our findings. Increased sphingomyelinase activity would lead to the increased release of ceramides stored in membrane sphingomyelins (Fig. 6). However, sphingomyelinase is known to release both CER[*NS*] and CER[*AS*] ceramides from sphingomyelins, in the *stratum corneum* at least [69], and CER[*AS*] ceramides remained unaltered by n-3PUFA supplementation in our study. We have previously shown that EPA, and its metabolite docosapentaenoic acid, can downregulate neutral sphingomyelinase and ceramide expression in rat brain [70]. DHA has previously been shown to downregulate ceramide release from sphingomyelins in retinal endothelial cells through a downregulation of acid sphingomyelinase [71]. However, the ceramide profile of the skin is unique and the effect of n-3PUFA on sphingomyelinases in the skin may be different.

Another potential store of ceramides in the skin is the glucosylceramides (Fig. 6). Although CER[*NS*] have been identified as being part of glucosylceramides in the epidermis, CER[*NDS*] ceramides have not to date [72]. The influence of n-3PUFA on the activity of glucosylceramidases, the enzymes that release ceramides from the glucosylceramides, is not known, and further studies would be needed to explore if increased glucosylceramidase activity could explain the increase of only CER[*NS*] and CER[*NDS*] species, as observed in our study. Inhibition of the uptake of ceramides into sphingomyelins or glucosylceramides could also explain our findings, although the regulation of these processes in skin is not yet understood [69], [72]. Overall, the impact of n-3PUFA on cutaneous sphingomyelins and glucosylceramides merits further investigation, as effects on the activity of specific sphingomyelinases or glucosylceramidases in our study cannot be ruled out.

A possible mechanism of increased ceramide production could be an effect on *de novo* ceramide biosynthesis (Fig. 6). CER[*NDS*] ceramides are produced upstream of other ceramides, and initially converted into CER[*NS*] species. DHA, but not EPA, has been found to block western-diet-induced upregulation of *de novo* ceramide synthesis in the liver, by blocking the induction of serine-palmitoyl transferase long chain base subunit-1 [73], but the unique sphingolipid metabolism in the skin could lead to different responses compared with other tissues.

The absence of any change in CER[*AS*], CER[*ADS*], CER[*AH*] or CER[*AP*] ceramides is notable, since it indicates that only ceramides containing a non-hydroxy fatty acid are affected by short-term n-3PUFA supplementation. There is evidence that n-3PUFA preferentially displace certain fatty acids from cell membranes, and ceramide-specific changes might be related to this [74]. Previous studies on the effect of n-3PUFA on ceramides in tissues other than skin have focussed on CER[*NS*] and CER[*NDS*] species [64], [65], [67], and so more studies are needed to explore this. The levels of epidermal CER[*EOS*], CER[*EOH*] and CER[*EOP*] ceramides were not altered either. These ceramide families are crucial in the formation of the epidermal barrier in the stratum corneum, and are unique in their requirement for LA [20]. LA deficiency results in substitution with oleic or other fatty acids instead [75]. Our results suggest that short-term n-3PUFA supplementation did not affect the availability of linoleic acid for continued synthesis of these species. However, since we have used a targeted approach to measure ceramides in this assay, we only evaluated a fixed panel of ceramide species. This means there could be new species formed following EPA or DHA supplementation that we did not identify. Examples of new species could include acyl-ceramides containing alpha-linolenic acid (ALA) instead of LA, should EPA or DHA undergo retroconversion to ALA (which has previously been observed in the blood [76]) in a similar manner that AA can undergo retroconversion to LA in the skin [77].

While we observed no change in the sphingoid bases or phosphorylated sphingoid bases measured following n-3PUFA supplementation, an increase in epidermal, although not dermal, C1P species following EPA treatment was noted. C1P species are synthesised from ceramides by ceramide kinase [61]. As we did not measure a significant increase in epidermal precursor ceramides, our findings could be attributed to increased ceramide kinase activity. Such enhanced activity could also explain the lack of increased levels of ceramides in the EPA-treated epidermis, as metabolism to C1P species would negate their accumulation (Fig. 6). Increased levels of C1P in the epidermis could have an anti-inflammatory effect, stimulating cell proliferation and migration, and inhibiting apoptosis [26], [61].

A valuable aspect of this study was the independent assessment of EPA and DHA, two n-3PUFA that are often considered together in nutritional studies (*e.g.* assessing intake of fish or fish oil). The different response of skin sphingolipids to EPA and DHA support the notion that their action relates to formation of specific lipid mediators, rather than n-3PUFA-related changes in membrane composition. For example, the endocannabinoid receptor CB1 is linked to ceramide synthesis through sphingomyelin hydrolysis and *de novo* synthesis [78], and we have previously shown that supplementation of *ex vivo* skin with EPA or DHA increased production of the endocannabinoid anandamide, as well as the EPA- and DHA-derived congeners [16]. Indeed, in an earlier study using the same *ex vivo* organ culture model we found that following just 3 days of supplementation with EPA or DHA the skin started to produce EPA- and DHA-derived eicosanoid and N-acyl ethanolamide species, respectively, in both the dermis and epidermis. This indicates that not only was the skin able to take up the PUFA and distribute them throughout the skin, but also metabolise the EPA and DHA into signalling compounds [16].

The different responses of the dermis and epidermis reported in this study could indicate compartmental differences in lipid metabolism. Whilst CER[*NS*] and CER[*NDS*] ceramides were increased in the dermis but not epidermis in response to EPA (Fig. 4), C1P species were increased in the epidermis but not the dermis (Fig. 5). In the skin organ culture method employed in this study, n-3PUFA supplementation was through the dermis that was submerged in culture medium, replicating the *in vivo* support of the epidermis. Skin was harvested after 6 days in culture, which we have previously shown is sufficient for dermal and epidermal uptake and metabolism of supplementary n-3PUFA [16] (and shown in Supplementary data S3). Therefore, differences in enzyme expression between the two skin compartments could contribute to different responses to n-3PUFA, although to-date the epidermis has been studied far more extensively than the dermis, and no comprehensive comparison of sphingolipid enzyme expression is available.

In light of differences seen in this study, an important aspect is the examination of the dermis and epidermis separately. We believe that this is the first investigation of the full ceramide profile and comparison of both skin layers. The different responses of the main skin compartments to n-3PUFA supplementation reveal the importance of studying full-thickness skin, since cross-talk and communication between the epidermal and dermal layers could have significant roles to play in skin health and disease [79]. The differences are highlighted when the proportions of ceramide families are compared with those found in previous studies that focussed on the stratum corneum [21]. Whilst tape-stripping of the stratum corneum is a useful, non-invasive sampling technique that allows analysis of the surface ceramides, it does not accurately reflect the profile of the dermis and epidermis, and biopsy sampling is the best approach to study full-thickness skin ceramides.

Overall, this *ex vivo* study has implications for our understanding of the role of n-3PUFA in skin health. The implications of EPA-induced ceramide changes in the skin should be explored further, and studies are needed to directly assess other aspects of skin physiology in this skin organ culture model following EPA supplementation, since we limited our analysis to ceramide profiling. Whilst the skin organ culture system attempts to replicate the systemic delivery and metabolism of n-3PUFA in the epidermis and dermis, clinical studies with dietary supplementation are needed to confirm whether their effects on cutaneous ceramides are reproducible *in vivo,* and allow the detailed examination of the impact of longer-term dietary supplementation on skin lipid biology.

The following are the supplementary data related to this article.

Table S1 MRM transitions and conditions for sphingolipid species analysis by UPLC/ESI-MS/MS. Table S1

Table S2 Concentrations of sphingolipid species in the dermis and epidermis, and their log2 fold change following supplementation with EPA and DHA. Data expressed as mean (*n* = 4). Table S2

Supplementary Fig. S3 Representative data showing the viability and metabolic competence of skin from an individual donor in *ex vivo* culture. Following an initial release of lactate dehydrogenase (LDH) into the culture medium following the trauma experienced by peripheral skin cells during biopsy sampling, LDH release settles within a day and remains low during culture (data are expressed as μUnits of LDH activity per ml culture medium). Neither EPA nor DHA affected viability and therefore LDH release (**A**). Skin biopsies cultured for 3 and 6 days with 50 μM EPA (green squares) or DHA (orange diamonds) show uptake of the fatty acids and alterations in the EPA and DHA content of the epidermis (**B**) and dermis (**C**), compared with vehicle (DMSO) treatment. EPA and DHA content are shown as % of total fatty acids in the epidermis (**B**) or dermis (**C**). Supplementary Fig. S3

## Author contributions

ACK designed experiments, performed experiments, analysed data and wrote the manuscript. MKT designed experiments, and performed experiments. LCB developed analytical tools. JLH conceived the study and wrote the manuscript. AN conceived the study, designed experiments, analysed data and wrote the manuscript.

## Funding

MK-T was supported by a research studentship awarded by the Manchester Pharmacy School. The project was supported in part by the Wellcome Trust (AN; project grant WT094028MA).

## Transparency document

Transparency document Image 1
