# Supplementary material for: Lipid functions in skin: Differential effects of n-3 polyunsaturated fatty acids on cutaneous ceramides, in a human skin organ culture model
Source: Biochim Biophys Acta. 2017 Sep;1859(9Part B):1679–89. doi: 10.1016/j.bbamem.2017.03.016 (PMC5504780; doi:10.1016/j.bbamem.2017.03.016)
Supplement: Table S1 — MRM transitions and conditions for sphingolipid species analysis by UPLC/ESI-MS/MS. [file mmc1.docx]

**Supplementary data S1**

**Table S1**: MRM transitions and conditions for sphingolipid species analysis by UPLC/ESI-MS/MS

| Species | Precursor  ion | Product  ion | Cone voltage (V) | Collision energy  (eV) | Indicative retention time (min) | |
| --- | --- | --- | --- | --- | --- | --- |
| Bases and phosphorylated species |  |  |  |  |  |  |
| C18 S | 300 | 282 | 25 | 21 | 8.64 |  |
| C18 DS | 302 | 284 | 25 | 21 | 8.80 |  |
| C18 S1P | 380 | 264 | 25 | 25 | 9.15 |  |
| C18 DS1P | 382 | 266 | 25 | 25 | 9.41 |  |
| N(14)S(18) C1P | 590 | 264 | 25 | 43 | 11.70 |  |
| N(16)S(18) C1P | 619 | 264 | 25 | 43 | 11.96 |  |
| N(16)DS(18) C1P | 621 | 266 | 25 | 46 | 12.02 |  |
| N(18)S(18) C1P | 647 | 264 | 25 | 48 | 12.45 |  |
| ADS | **[M+H]^+^** | **[M+H-FA-H_2_O]^+^** |  |  |  |  |
| A(18)DS(20) | 612.573 | 312 | 30 | 30 | 11.20 |  |
| A(16)DS(22) | 612.573 | 340 | 30 | 30 | 11.19 |  |
| A(24)DS(16) | 640.625 | 256 | 30 | 30 | 11.75 |  |
| A(20)DS(20) | 640.625 | 312 | 30 | 30 | 11.50 |  |
| A(18)DS(22) | 640.625 | 340 | 30 | 30 | 11.51 |  |
| A(16)DS(24) | 640.625 | 368 | 30 | 30 | 11.60 |  |
| A(24)DS(17) | 654.640 | 270 | 30 | 30 | 11.97 |  |
| A(23)DS(18) | 654.640 | 284 | 30 | 30 | 12.32 |  |
| A(22)DS(19) | 654.640 | 298 | 30 | 30 | 11.65 |  |
| A(18)DS(23) | 654.640 | 354 | 30 | 30 | 11.68 |  |
| A(16)DS(25) | 654.640 | 382 | 30 | 30 | 11.96 |  |
| A(26)DS(16) | 668.656 | 256 | 30 | 30 | 11.91 |  |
| A(24)DS(18) | 668.656 | 284 | 30 | 30 | 12.12 |  |
| A(18)DS(24) | 668.656 | 368 | 30 | 30 | 11.91 |  |
| A(16)DS(26) | 668.656 | 396 | 30 | 30 | 11.98 |  |
| A(27)DS(16) | 682.672 | 256 | 30 | 30 | 12.16 |  |
| A(25)DS(18) | 682.672 | 284 | 30 | 30 | 12.41 |  |
| A(24)DS(19) | 682.672 | 298 | 30 | 30 | 12.34 |  |
| A(23)DS(20) | 682.672 | 312 | 30 | 30 | 12.81 |  |
| A(26)DS(18) | 696.687 | 284 | 30 | 30 | 12.65 |  |
| A(24)DS(20) | 696.687 | 312 | 30 | 30 | 12.60 |  |
| A(22)DS(22) | 696.687 | 340 | 30 | 30 | 12.28 |  |
| A(26)DS(19) | 710.703 | 298 | 30 | 30 | 12.87 |  |
| A(25)DS(20) | 710.703 | 312 | 30 | 30 | 13.40 |  |
| A(24)DS(21) | 710.703 | 326 | 30 | 30 | 13.34 |  |
| A(23)DS(22) | 710.703 | 340 | 30 | 30 | 13.34 |  |
| A(26)DS(20) | 724.719 | 312 | 30 | 30 | 13.13 |  |
| A(24)DS(22) | 724.719 | 340 | 30 | 30 | 13.11 |  |
| AH | **[M+H-H_2_O]^+^** | **[M+H-FA-2H_2_O]^+^** |  |  |  |  |
| A(24)H(14) | 608.562 | 224 | 30 | 30 | 10.47 |  |
| A(22)H(16) | 608.562 | 252 | 30 | 30 | 10.81 |  |
| A(20)H(18) | 608.562 | 280 | 30 | 30 | 10.93 |  |
| A(25)H(14) | 622.577 | 224 | 30 | 30 | 10.68 |  |
| A(24)H(15) | 622.577 | 238 | 30 | 30 | 10.82 |  |
| A(23)H(16) | 622.577 | 252 | 30 | 30 | 11.07 |  |
| A(22)H(17) | 622.577 | 266 | 30 | 30 | 11.47 |  |
| A(26)H(14) | 636.593 | 224 | 30 | 30 | 11.10 |  |
| A(24)H(16) | 636.593 | 252 | 30 | 30 | 11.10 |  |
| A(22)H(18) | 636.593 | 280 | 30 | 30 | 11.08 |  |
| A(26)H(15) | 650.609 | 238 | 30 | 30 | 11.20 |  |
| A(25)H(16) | 650.609 | 252 | 30 | 30 | 11.25 |  |
| A(24)H(17) | 650.609 | 266 | 30 | 30 | 11.22 |  |
| A(28)H(14) | 664.624 | 224 | 30 | 30 | 11.40 |  |
| A(26)H(16) | 664.624 | 252 | 30 | 30 | 11.42 |  |
| A(24)H(18) | 664.624 | 280 | 30 | 30 | 11.39 |  |
| A(28)H(15) | 678.64 | 238 | 30 | 30 | 11.55 |  |
| A(27)H(16) | 678.64 | 252 | 30 | 30 | 11.52 |  |
| A(26)H(17) | 678.64 | 266 | 30 | 30 | 11.57 |  |
| A(25)H(18) | 678.64 | 280 | 30 | 30 | 11.52 |  |
| A(24)H(19) | 678.64 | 294 | 30 | 30 | 11.50 |  |
| A(28)H(16) | 692.655 | 252 | 30 | 30 | 11.72 |  |
| A(26)H(18) | 692.655 | 280 | 30 | 30 | 11.73 |  |
| A(24)H(20) | 692.655 | 308 | 30 | 30 | 11.63 |  |
| A(28)H(17) | 706.671 | 266 | 30 | 30 | 11.97 |  |
| A(27)H(18) | 706.671 | 280 | 30 | 30 | 11.91 |  |
| A(26)H(19) | 706.671 | 294 | 30 | 30 | 11.88 |  |
| A(25)H(20) | 706.671 | 308 | 30 | 30 | 11.86 |  |
| A(28)H(18) | 720.687 | 280 | 30 | 30 | 12.16 |  |
| A(26)H(20) | 720.687 | 308 | 30 | 30 | 12.06 |  |
| A(24)H(22) | 720.687 | 336 | 30 | 30 | 12.01 |  |
| A(29)H(18) | 734.702 | 280 | 30 | 30 | 12.35 |  |
| A(28)H(19) | 734.702 | 294 | 30 | 30 | 12.35 |  |
| A(27)H(20) | 734.702 | 308 | 30 | 30 | 12.30 |  |
| A(26)H(21) | 734.702 | 322 | 30 | 30 | 12.27 |  |
| A(30)H(18) | 748.718 | 280 | 30 | 30 | 12.56 |  |
| A(28)H(20) | 748.718 | 308 | 30 | 30 | 12.56 |  |
| A(26)H(22) | 748.718 | 336 | 30 | 30 | 12.50 |  |
| AP | **[M+H]^+^** | **[M+H-FA-2H_2_O]^+^** |  |  |  |  |
| A(22)P(16) | 628.588 | 254 | 30 | 30 | 11.10 |  |
| A(24)P(16) | 656.620 | 254 | 30 | 30 | 11.41 |  |
| A(22)P(18) | 656.620 | 282 | 30 | 30 | 11.37 |  |
| A(20)P(20) | 656.620 | 310 | 30 | 30 | 11.32 |  |
| A(24)P(17) | 670.635 | 268 | 30 | 30 | 11.56 |  |
| A(22)P(19) | 670.635 | 296 | 30 | 30 | 11.50 |  |
| A(26)P(16) | 684.651 | 254 | 30 | 30 | 11.80 |  |
| A(24)P(18) | 684.651 | 282 | 30 | 30 | 11.75 |  |
| A(22)P(20) | 684.651 | 310 | 30 | 30 | 11.68 |  |
| A(26)P(17) | 698.666 | 268 | 30 | 30 | 11.97 |  |
| A(25)P(18) | 698.666 | 282 | 30 | 30 | 11.94 |  |
| A(24)P(19) | 698.666 | 296 | 30 | 30 | 11.90 |  |
| A(26)P(18) | 712.682 | 282 | 30 | 30 | 12.16 |  |
| A(24)P(20) | 712.682 | 310 | 30 | 30 | 12.10 |  |
| A(26)P(19) | 726.698 | 324 | 30 | 30 | 12.32 |  |
| A(25)P(20) | 726.698 | 296 | 30 | 30 | 12.36 |  |
| A(24)P(21) | 726.698 | 310 | 30 | 30 | 12.33 |  |
| A(26)P(20) | 740.713 | 310 | 30 | 30 | 12.58 |  |
| A(24)P(22) | 740.713 | 338 | 30 | 30 | 12.81 |  |
| A(26)P(22) | 754.729 | 338 | 30 | 30 | 11.62 |  |
| AS | **[M+H]^+^** | **[M+H-FA-2H_2_O]^+^** |  |  |  |  |
| A(25)S(16) | 652.625 | 236 | 30 | 30 | 11.84 |  |
| A(24)S(17) | 652.625 | 250 | 30 | 30 | 11.80 |  |
| A(23)S(18) | 652.625 | 264 | 30 | 30 | 12.19 |  |
| A(26)S(16) | 666.640 | 236 | 30 | 30 | 12.03 |  |
| A(24)S(18) | 666.640 | 264 | 30 | 30 | 11.97 |  |
| A(27)S(16) | 680.656 | 236 | 30 | 30 | 12.27 |  |
| A(26)S(17) | 680.656 | 250 | 30 | 30 | 12.24 |  |
| A(25)S(18) | 680.656 | 264 | 30 | 30 | 12.20 |  |
| A(24)S(19) | 680.656 | 278 | 30 | 30 | 12.17 |  |
| A(23)S(20) | 680.656 | 292 | 30 | 30 | 12.15 |  |
| A(22)S(21) | 680.656 | 306 | 30 | 30 | 12.27 |  |
| A(26)S(18) | 694.672 | 264 | 30 | 30 | 12.44 |  |
| A(24)S(20) | 694.672 | 292 | 30 | 30 | 12.39 |  |
| A(27)S(18) | 708.687 | 264 | 30 | 30 | 12.70 |  |
| A(26)S(19) | 708.687 | 278 | 30 | 30 | 12.67 |  |
| A(24)S(21) | 708.687 | 306 | 30 | 30 | 12.62 |  |
| A(23)S(22) | 708.687 | 320 | 30 | 30 | 12.63 |  |
| A(26)S(20) | 722.703 | 292 | 30 | 30 | 12.92 |  |
| A(24)S(22) | 722.703 | 320 | 30 | 30 | 12.88 |  |
| A(28)S(19) | 736.718 | 278 | 30 | 30 | 13.28 |  |
| A(25)S(22) | 736.718 | 320 | 30 | 30 | 13.20 |  |
| A(26)S(22) | 750.734 | 320 | 30 | 30 | 13.49 |  |
| NDS | **[M+H]^+^** | **[M+H-FA-2H_2_O]^+^** |  |  |  |  |
| N(24)DS(16) | 624.630 | 256 | 30 | 30 | 11.92 |  |
| N(22)DS(18) | 624.630 | 284 | 30 | 30 | 11.86 |  |
| N(20)DS(20) | 624.630 | 312 | 30 | 30 | 11.84 |  |
| N(18)DS(22) | 624.630 | 340 | 30 | 30 | 11.87 |  |
| N(16)DS(24) | 624.630 | 368 | 30 | 30 | 11.90 |  |
| N(25)DS(16) | 638.646 | 256 | 30 | 30 | 12.09 |  |
| N(24)DS(17) | 638.646 | 270 | 30 | 30 | 12.15 |  |
| N(23)DS(18) | 638.646 | 284 | 30 | 30 | 12.07 |  |
| N(22)DS(19) | 638.646 | 298 | 30 | 30 | 12.06 |  |
| N(26)DS(16) | 652.661 | 256 | 30 | 30 | 12.34 |  |
| N(24)DS(18) | 652.661 | 284 | 30 | 30 | 12.31 |  |
| N(20)DS(22) | 652.661 | 340 | 30 | 30 | 12.28 |  |
| N(18)DS(24) | 652.661 | 368 | 30 | 30 | 12.32 |  |
| N(16)DS(26) | 652.661 | 396 | 30 | 30 | 12.35 |  |
| N(26)DS(17) | 666.677 | 270 | 30 | 30 | 12.61 |  |
| N(25)DS(18) | 666.677 | 284 | 30 | 30 | 12.55 |  |
| N(24)DS(19) | 666.677 | 298 | 30 | 30 | 12.56 |  |
| N(23)DS(20) | 666.677 | 312 | 30 | 30 | 12.52 |  |
| N(26)DS(18) | 680.692 | 284 | 30 | 30 | 12.86 |  |
| N(24)DS(20) | 680.692 | 312 | 30 | 30 | 12.79 |  |
| N(22)DS(22) | 680.692 | 340 | 30 | 30 | 12.76 |  |
| N(20)DS(24) | 680.692 | 368 | 30 | 30 | 12.79 |  |
| N(18)DS(26) | 680.692 | 396 | 30 | 30 | 12.86 |  |
| N(28)DS(17) | 694.708 | 270 | 30 | 30 | 13.21 |  |
| N(27)DS(18) | 694.708 | 284 | 30 | 30 | 13.16 |  |
| N(26)DS(19) | 694.708 | 298 | 30 | 30 | 13.11 |  |
| N(25)DS(20) | 694.708 | 312 | 30 | 30 | 13.08 |  |
| N(24)DS(21) | 694.708 | 326 | 30 | 30 | 13.08 |  |
| N(23)DS(22) | 694.708 | 340 | 30 | 30 | 13.08 |  |
| N(22)DS(23) | 694.708 | 354 | 30 | 30 | 13.04 |  |
| N(28)DS(18) | 708.724 | 284 | 30 | 30 | 13.48 |  |
| N(26)DS(20) | 708.724 | 312 | 30 | 30 | 13.40 |  |
| N(24)DS(22) | 708.724 | 340 | 30 | 30 | 13.36 |  |
| N(22)DS(24) | 708.724 | 368 | 30 | 30 | 13.36 |  |
| N(20)DS(26) | 708.724 | 396 | 30 | 30 | 13.40 |  |
| N(28)DS(19) | 722.739 | 298 | 30 | 30 | 13.77 |  |
| N(27)DS(20) | 722.739 | 312 | 30 | 30 | 13.75 |  |
| N(26)DS(21) | 722.739 | 326 | 30 | 30 | 13.72 |  |
| N(25)DS(22) | 722.739 | 340 | 30 | 30 | 13.68 |  |
| N(24)DS(23) | 722.739 | 354 | 30 | 30 | 13.66 |  |
| N(23)DS(24) | 722.739 | 368 | 30 | 30 | 13.67 |  |
| N(22)DS(25) | 722.739 | 382 | 30 | 30 | 13.69 |  |
| N(28)DS(20) | 736.755 | 312 | 30 | 30 | 14.10 |  |
| N(26)DS(22) | 736.755 | 340 | 30 | 30 | 14.03 |  |
| N(24)DS(24) | 736.755 | 368 | 30 | 30 | 14.02 |  |
| N(22)DS(26) | 736.755 | 396 | 30 | 30 | 14.03 |  |
| N(29)DS(20) | 750.771 | 312 | 30 | 35 | 14.49 |  |
| N(28)DS(21) | 750.771 | 326 | 30 | 35 | 14.43 |  |
| N(27)DS(22) | 750.771 | 340 | 30 | 35 | 14.41 |  |
| N(26)DS(23) | 750.771 | 354 | 30 | 35 | 14.38 |  |
| N(25)DS(24) | 750.771 | 368 | 30 | 35 | 14.38 |  |
| N(24)DS(25) | 750.771 | 382 | 30 | 35 | 14.37 |  |
| N(23)DS(26) | 750.771 | 396 | 30 | 35 | 14.40 |  |
| N(22)DS(27) | 750.771 | 410 | 30 | 35 | 14.41 |  |
| N(30)DS(20) | 764.786 | 312 | 30 | 35 | 14.87 |  |
| N(28)DS(22) | 764.786 | 340 | 30 | 35 | 14.81 |  |
| N(26)DS(24) | 764.786 | 368 | 30 | 35 | 14.76 |  |
| N(24)DS(26) | 764.786 | 396 | 30 | 35 | 14.76 |  |
| N(30)DS(21) | 778.802 | 326 | 30 | 35 | 15.26 |  |
| N(29)DS(22) | 778.802 | 340 | 30 | 35 | 14.21 |  |
| N(28)DS(23) | 778.802 | 354 | 30 | 35 | 15.22 |  |
| N(27)DS(24) | 778.802 | 368 | 30 | 35 | 15.19 |  |
| N(26)DS(25) | 778.802 | 382 | 30 | 35 | 15.17 |  |
| N(25)DS(26) | 778.802 | 396 | 30 | 35 | 15.16 |  |
| N(24)DS(27) | 778.802 | 410 | 30 | 35 | 15.19 |  |
| N(30)DS(22) | 792.818 | 340 | 30 | 35 | 14.54 |  |
| N(28)DS(24) | 792.818 | 368 | 30 | 35 | 14.65 |  |
| N(26)DS(26) | 792.818 | 396 | 30 | 35 | 14.84 |  |
| N(29)DS(24) | 806.833 | 368 | 30 | 35 | 14.96 |  |
| N(28)DS(25) | 806.833 | 382 | 30 | 35 | 15.00 |  |
| N(27)DS(26) | 806.833 | 396 | 30 | 35 | 15.03 |  |
| N(26)DS(27) | 806.833 | 410 | 30 | 35 | 15.70 |  |
| N(30)DS(24) | 820.849 | 368 | 30 | 35 | 15.32 |  |
| N(28)DS(26) | 820.849 | 396 | 30 | 35 | 15.66 |  |
| N(26)DS(28) | 820.849 | 424 | 30 | 35 | 15.66 |  |
| NH | **[M+H-H_2_O]^+^** | **[M+H-FA-2H_2_O]^+^** |  |  |  |  |
| N(24)H(16) | 620.597 | 224 | 30 | 30 | 11.28 |  |
| N(26)H(14) | 620.597 | 252 | 30 | 30 | 11.26 |  |
| N(26)H(15) | 634.614 | 238 | 30 | 30 | 11.44 |  |
| N(25)H(16) | 634.614 | 252 | 30 | 30 | 11.44 |  |
| N(24)H(17) | 634.614 | 266 | 30 | 30 | 11.40 |  |
| N(23)H(18) | 634.614 | 280 | 30 | 30 | 11.37 |  |
| N(26)H(16) | 648.629 | 252 | 30 | 30 | 11.57 |  |
| N(24)H(18) | 648.629 | 280 | 30 | 30 | 11.54 |  |
| N(26)H(17) | 662.645 | 266 | 30 | 30 | 11.79 |  |
| N(25)H(18) | 662.645 | 280 | 30 | 30 | 11.75 |  |
| N(28)H(16) | 676.661 | 252 | 30 | 30 | 11.94 |  |
| N(26)H(18) | 676.661 | 280 | 30 | 30 | 11.94 |  |
| N(24)H(20) | 676.661 | 308 | 30 | 30 | 11.87 |  |
| N(28)H(17) | 690.676 | 266 | 30 | 30 | 12.24 |  |
| N(27)H(18) | 690.676 | 280 | 30 | 30 | 12.17 |  |
| N(26)H(19) | 690.676 | 294 | 30 | 30 | 12.14 |  |
| N(25)H(20) | 690.676 | 308 | 30 | 30 | 12.09 |  |
| N(28)H(18) | 704.692 | 280 | 30 | 30 | 12.41 |  |
| N(26)H(20) | 704.692 | 308 | 30 | 30 | 12.34 |  |
| N(28)H(19) | 718.707 | 294 | 30 | 30 | 12.63 |  |
| N(27)H(20) | 718.707 | 308 | 30 | 30 | 12.60 |  |
| N(26)H(21) | 718.707 | 322 | 30 | 30 | 12.55 |  |
| N(30)H(19) | 732.723 | 280 | 30 | 30 | 12.96 |  |
| N(28)H(20) | 732.723 | 308 | 30 | 30 | 12.86 |  |
| N(26)H(22) | 732.723 | 336 | 30 | 30 | 12.79 |  |
| N(30)H(19) | 746.739 | 294 | 30 | 35 | 13.21 |  |
| N(29)H(20) | 746.739 | 308 | 30 | 35 | 13.15 |  |
| N(28)H(21) | 746.739 | 322 | 30 | 35 | 13.13 |  |
| N(30)H(20) | 760.754 | 308 | 30 | 35 | 13.47 |  |
| N(28)H(22) | 760.754 | 336 | 30 | 35 | 13.41 |  |
| NP | **[M+H]^+^** | **[M+H-FA-2H_2_O]^+^** |  |  |  |  |
| N(24)P(16) | 640.625 | 254 | 30 | 30 | 11.59 |  |
| N(24)P(17) | 654.640 | 268 | 30 | 30 | 11.72 |  |
| N(26)P(16) | 668.656 | 254 | 30 | 30 | 11.97 |  |
| N(24)P(18) | 668.656 | 282 | 30 | 30 | 11.91 |  |
| N(26)P(17) | 682.672 | 268 | 30 | 30 | 12.17 |  |
| N(25)P(18) | 682.672 | 282 | 30 | 30 | 12.13 |  |
| N(24)P(19) | 682.672 | 296 | 30 | 30 | 12.05 |  |
| N(23)P(20) | 682.672 | 310 | 30 | 30 | 12.06 |  |
| N(28)P(16) | 696.687 | 254 | 30 | 30 | 12.48 |  |
| N(26)P(18) | 696.687 | 282 | 30 | 30 | 12.37 |  |
| N(24)P(20) | 696.687 | 310 | 30 | 30 | 12.31 |  |
| N(28)P(17) | 710.703 | 268 | 30 | 30 | 12.67 |  |
| N(27)P(18) | 710.703 | 282 | 30 | 30 | 12.64 |  |
| N(26)P(19) | 710.703 | 296 | 30 | 30 | 12.58 |  |
| N(25)P(20) | 710.703 | 310 | 30 | 30 | 12.55 |  |
| N(24)P(21) | 710.703 | 324 | 30 | 30 | 12.51 |  |
| N(28)P(18) | 724.718 | 282 | 30 | 30 | 12.90 |  |
| N(26)P(20) | 724.718 | 310 | 30 | 30 | 12.83 |  |
| N(24)P(22) | 724.718 | 338 | 30 | 30 | 12.76 |  |
| N(26)P(21) | 738.734 | 324 | 30 | 30 | 13.09 |  |
| N(25)P(22) | 738.734 | 338 | 30 | 30 | 13.07 |  |
| N(28)P(20) | 752.750 | 310 | 30 | 35 | 13.45 |  |
| N(26)P(22) | 752.750 | 338 | 30 | 35 | 13.39 |  |
| N(24)P(24) | 752.750 | 366 | 30 | 35 | 13.36 |  |
| N(28)P(21) | 766.765 | 324 | 30 | 35 | 13.76 |  |
| N(27)P(22) | 766.765 | 338 | 30 | 35 | 13.72 |  |
| N(26)P(23) | 766.765 | 352 | 30 | 35 | 13.71 |  |
| N(30)P(20) | 780.781 | 310 | 30 | 35 | 14.14 |  |
| N(28)P(22) | 780.781 | 338 | 30 | 35 | 14.06 |  |
| N(26)P(24) | 780.781 | 366 | 30 | 35 | 14.02 |  |
| N(24)P(26) | 780.781 | 394 | 30 | 35 | 14.00 |  |
| N(30)P(21) | 794.797 | 324 | 30 | 35 | 14.50 |  |
| N(29)P(22) | 794.797 | 338 | 30 | 35 | 14.43 |  |
| N(28)P(23) | 794.797 | 352 | 30 | 35 | 14.40 |  |
| N(27)P(24) | 794.797 | 366 | 30 | 35 | 14.40 |  |
| N(26)P(25) | 794.797 | 380 | 30 | 35 | 14.35 |  |
| N(30)P(22) | 808.812 | 338 | 30 | 35 | 14.86 |  |
| N(28)P(24) | 808.812 | 366 | 30 | 35 | 14.80 |  |
| N(26)P(26) | 808.812 | 394 | 30 | 35 | 14.74 |  |
| NS | **[M+H]^+^** | **[M+H-FA-2H_2_O]^+^** |  |  |  |  |
| N(24)S(16) | 622.614 | 236 | 30 | 30 | 11.81 |  |
| N(22)S(18) | 622.614 | 264 | 30 | 30 | 11.77 |  |
| N(25)S(16) | 636.630 | 236 | 30 | 30 | 12.02 |  |
| N(24)S(17) | 636.630 | 250 | 30 | 30 | 11.99 |  |
| N(23)S(18) | 636.630 | 264 | 30 | 30 | 11.97 |  |
| N(22)S(19) | 636.630 | 278 | 30 | 30 | 11.93 |  |
| N(21)S(20) | 636.630 | 292 | 30 | 30 | 11.92 |  |
| N(26)S(16) | 650.645 | 236 | 30 | 30 | 12.27 |  |
| N(24)S(18) | 650.645 | 264 | 30 | 30 | 12.19 |  |
| N(22)S(20) | 650.645 | 292 | 30 | 30 | 12.16 |  |
| N(20)S(22) | 650.645 | 320 | 30 | 30 | 12.13 |  |
| N(27)S(16) | 664.661 | 236 | 30 | 30 | 12.51 |  |
| N(26)S(17) | 664.661 | 250 | 30 | 30 | 12.48 |  |
| N(24)S(19) | 664.661 | 278 | 30 | 30 | 12.38 |  |
| N(23)S(20) | 664.661 | 292 | 30 | 30 | 12.39 |  |
| N(22)S(21) | 664.661 | 306 | 30 | 30 | 12.38 |  |
| N(28)S(16) | 678.677 | 236 | 30 | 30 | 12.82 |  |
| N(26)S(18) | 678.677 | 264 | 30 | 30 | 12.71 |  |
| N(24)S(20) | 678.677 | 292 | 30 | 30 | 12.65 |  |
| N(28)S(17) | 692.692 | 250 | 30 | 30 | 13.04 |  |
| N(27)S(18) | 692.692 | 264 | 30 | 30 | 12.99 |  |
| N(26)S(19) | 692.692 | 278 | 30 | 30 | 12.95 |  |
| N(25)S(20) | 692.692 | 292 | 30 | 30 | 12.93 |  |
| N(24)S(21) | 692.692 | 306 | 30 | 30 | 12.89 |  |
| N(23)S(22) | 692.692 | 320 | 30 | 30 | 12.90 |  |
| N(28)S(18) | 706.708 | 264 | 30 | 30 | 13.30 |  |
| N(26)S(20) | 706.708 | 292 | 30 | 30 | 13.22 |  |
| N(24)S(22) | 706.708 | 320 | 30 | 30 | 13.17 |  |
| N(22)S(24) | 706.708 | 348 | 30 | 30 | 13.18 |  |
| N(29)S(18) | 720.724 | 264 | 30 | 30 | 13.63 |  |
| N(28)S(19) | 720.724 | 278 | 30 | 30 | 13.59 |  |
| N(27)S(20) | 720.724 | 292 | 30 | 30 | 13.53 |  |
| N(26)S(21) | 720.724 | 306 | 30 | 30 | 13.52 |  |
| N(25)S(22) | 720.724 | 320 | 30 | 30 | 13.49 |  |
| N(24)S(23) | 720.724 | 334 | 30 | 30 | 13.48 |  |
| N(23)S(24) | 720.724 | 348 | 30 | 30 | 13.48 |  |
| N(28)S(20) | 734.739 | 292 | 30 | 30 | 13.90 |  |
| N(26)S(22) | 734.739 | 320 | 30 | 30 | 13.83 |  |
| N(24)S(24) | 734.739 | 348 | 30 | 30 | 13.80 |  |
| N(29)S(20) | 748.755 | 292 | 30 | 30 | 14.25 |  |
| N(28)S(21) | 748.755 | 306 | 30 | 30 | 14.25 |  |
| N(27)S(22) | 748.755 | 320 | 30 | 30 | 14.18 |  |
| N(26)S(23) | 748.755 | 334 | 30 | 30 | 14.16 |  |
| N(25)S(24) | 748.755 | 348 | 30 | 30 | 14.17 |  |
| N(24)S(25) | 748.755 | 362 | 30 | 30 | 14.13 |  |
| N(30)S(20) | 762.771 | 292 | 30 | 30 | 14.64 |  |
| N(28)S(22) | 762.771 | 320 | 30 | 30 | 14.59 |  |
| N(26)S(24) | 762.771 | 348 | 30 | 30 | 14.53 |  |
| N(24)S(26) | 762.771 | 376 | 30 | 30 | 14.50 |  |
| N(30)S(21) | 776.786 | 306 | 30 | 30 | 15.04 |  |
| N(29)S(22) | 776.786 | 320 | 30 | 30 | 15.00 |  |
| N(28)S(23) | 776.786 | 334 | 30 | 30 | 14.94 |  |
| N(26)S(25) | 776.786 | 362 | 30 | 30 | 14.90 |  |
| N(25)S(26) | 776.786 | 376 | 30 | 30 | 14.90 |  |
| N(30)S(22) | 790.802 | 320 | 30 | 30 | 15.40 |  |
| N(28)S(24) | 790.802 | 348 | 30 | 30 | 15.34 |  |
| N(26)S(26) | 790.802 | 376 | 30 | 30 | 15.33 |  |
| EOH | **[M+H-H_2_O]^+^** | **[M+H-FA-2H_2_O]^+^** |  |  |  |  |
| E(18:2)O(30)H(18) | 1010.948 | 280 | 30 | 40 | 15.57 |  |
| E(18:2)O(28)H(20) | 1010.948 | 308 | 30 | 40 | 15.43 |  |
| E(18:2)O(31)H(18) | 1024.963 | 280 | 30 | 40 | 15.98 |  |
| E(18:2)O(30)H(19) | 1024.963 | 294 | 30 | 40 | 15.89 |  |
| E(18:2)O(29)H(20) | 1024.963 | 308 | 30 | 40 | 15.86 |  |
| E(18:2)O(32)H(18) | 1038.979 | 280 | 30 | 40 | 16.44 |  |
| E(18:2)O(30)H(20) | 1038.979 | 308 | 30 | 40 | 16.30 |  |
| E(18:2)O(32)H(19) | 1052.995 | 294 | 30 | 40 | 16.79 |  |
| E(18:2)O(31)H(20) | 1052.995 | 308 | 30 | 40 | 16.73 |  |
| E(18:2)O(30)H(21) | 1052.995 | 322 | 30 | 40 | 16.65 |  |
| E(18:2)O(32)H(20) | 1067.01 | 308 | 30 | 40 | 17.19 |  |
| E(18:2)O(30)H(22) | 1067.01 | 336 | 30 | 40 | 17.05 |  |
| EOP | **[M+H]^+^** | **[M+H-FA-2H_2_O]^+^** |  |  |  |  |
| E(18:2)O(32)P(18) | 1059.006 | 282 | 30 | 45 | 17.32 |  |
| E(18:2)O(30)P(20) | 1059.006 | 310 | 30 | 45 | 17.14 |  |
| E(18:2)O(28)P(22) | 1059.006 | 338 | 30 | 45 | 17.04 |  |
| E(18:2)O(31)P(20) | 1073.021 | 310 | 30 | 45 | 17.65 |  |
| E(18:2)O(30)P(21) | 1073.021 | 324 | 30 | 45 | 17.57 |  |
| E(18:2)O(29)P(22) | 1073.021 | 338 | 30 | 45 | 17.55 |  |
| E(18:2)O(32)P(20) | 1087.037 | 310 | 30 | 45 | 18.13 |  |
| E(18:2)O(30)P(22) | 1087.037 | 338 | 30 | 45 | 18.03 |  |
| EOS | **[M+H]^+^** | **[M+H-FA-2H_2_O]^+^** |  |  |  |  |
| E(18:2)O(30)S(18) | 1012.964 | 264 | 30 | 45 | 16.97 |  |
| E(18:2)O(30)S(19) | 1026.979 | 278 | 30 | 45 | 17.38 |  |
| E(18:2)O(29)S(20) | 1026.979 | 292 | 30 | 45 | 17.30 |  |
| E(18:2)O(30)S(20) | 1040.995 | 292 | 30 | 45 | 17.77 |  |
| E(18:2)O(28)S(22) | 1040.995 | 320 | 30 | 45 | 17.68 |  |
| E(18:2)O(32)S(19) | 1055.011 | 278 | 30 | 45 | 18.35 |  |
| E(18:2)O(31)S(20) | 1055.011 | 292 | 30 | 45 | 18.27 |  |
| E(18:2)O(30)S(21) | 1055.011 | 306 | 30 | 45 | 18.19 |  |
| E(18:2)O(32)S(20) | 1069.026 | 292 | 30 | 45 | 18.78 |  |
| E(18:2)O(30)S(22) | 1069.026 | 320 | 30 | 45 | 18.65 |  |
