# Supplementary material for: Lipid functions in skin: Differential effects of n-3 polyunsaturated fatty acids on cutaneous ceramides, in a human skin organ culture model
Source: Biochim Biophys Acta. 2017 Sep;1859(9Part B):1679–89. doi: 10.1016/j.bbamem.2017.03.016 (PMC5504780; doi:10.1016/j.bbamem.2017.03.016)
Supplement: Table S2 — Concentrations of sphingolipid species in the dermis and epidermis, and their log2 fold change following supplementation with EPA and DHA. Data expressed as mean (n = 4). [file mmc2.docx]

**Supplementary data S2**

**Table S2**: Concentrations of sphingolipid species in the dermis and epidermis, and their log2 fold change following supplementation with EPA and DHA. Data expressed as mean (n=4).

| **Species** | **Concentration (pmol/g protein)** | | | | | | **Log2FC vs control** | | | |
| --- | --- | --- | --- | --- | --- | --- | --- | --- | --- | --- |
|  | **Dermis** | | | **Epidermis** | | | **Dermis** | | **Epidermis** | |
|  | **Control** | **EPA** | **DHA** | **Control** | **EPA** | **DHA** | **EPA** | **DHA** | **EPA** | **DHA** |
| **Bases and phosphorylated species** |  |  |  |  |  |  |  |  |  |  |
| **C18 S** | 1390.66 | 1339.82 | 1387.38 | 14629.09 | 13180.30 | 12352.79 | -0.06 | 0.00 | -0.23 | -0.29 |
| **C18 DS** | 64.10 | 55.14 | 55.38 | 450.43 | 439.93 | 458.04 | -0.13 | -0.15 | -0.02 | 0.03 |
| **C18 S1P** | 197.81 | 193.16 | 183.58 | 363.36 | 375.18 | 404.32 | -0.04 | 0.19 | 0.10 | 0.20 |
| **C18 DS1P** | 0.00 | 0.00 | 49.91 | 0.00 | 0.00 | 27.94 | - | - | - | - |
| **N(14)S(18) C1P** | 5.31 | 10.34 | 16.54 | 3.55 | 24.69 | 36.72 | - | - | - | - |
| **N(16)S(18) C1P** | 4350.96 | 3717.14 | 3292.42 | 2147.04 | 5077.57 | 2512.12 | 0.13 | -0.17 | 1.16 | 0.24 |
| **N(16)DS(18) C1P** | 561.26 | 706.85 | 774.16 | 229.91 | 329.42 | 220.43 | -0.20 | 0.00 | 0.54 | -0.05 |
| **N(18)S(18) C1P** | 17107.65 | 16290.16 | 12564.78 | 16324.00 | 28991.57 | 17803.45 | 0.17 | -0.19 | 0.95 | 0.20 |
| **ADS** |  |  |  |  |  |  |  |  |  |  |
| **A(18)DS(20)** | 4.87 | 4.10 | 4.35 | 6.64 | 15.42 | 11.41 | -0.22 | -0.06 | 1.19 | 0.76 |
| **A(16)DS(22)** | 3.04 | 2.34 | 1.86 | 4.09 | 4.83 | 4.01 | -0.41 | -0.77 | - | - |
| **A(24)DS(16)** | 0.00 | 0.32 | 0.52 | 9.14 | 14.53 | 13.15 | - | - | 0.68 | - |
| **A(20)DS(20)** | 1.54 | 1.35 | 1.42 | 8.93 | 8.99 | 11.48 | - | - | 0.01 | 0.30 |
| **A(18)DS(22)** | 44.95 | 47.22 | 50.81 | 103.41 | 141.79 | 147.60 | -0.02 | 0.15 | 0.42 | 0.47 |
| **A(16)DS(24)** | 15.82 | 12.38 | 13.65 | 145.29 | 152.41 | 177.79 | 0.07 | 0.17 | 0.12 | 0.22 |
| **A(24)DS(17)** | 4.07 | 2.96 | 3.68 | 21.06 | 37.99 | 31.25 | -0.23 | -0.11 | 0.76 | 0.65 |
| **A(23)DS(18)** | 42.59 | 40.40 | 36.24 | 64.88 | 77.29 | 102.29 | 0.04 | -0.09 | 0.34 | 0.71 |
| **A(22)DS(19)** | 1.19 | 0.48 | 0.62 | 2.28 | 4.29 | 2.61 | - | - | - | - |
| **A(18)DS(23)** | 9.57 | 4.47 | 5.81 | 27.24 | 36.22 | 36.80 | - | - | 0.53 | 0.49 |
| **A(16)DS(25)** | 2.93 | 2.44 | 2.96 | 31.73 | 50.18 | 35.12 | 0.12 | 0.13 | 0.32 | 0.09 |
| **A(26)DS(16)** | 4.10 | 3.17 | 3.68 | 31.86 | 46.58 | 35.08 | - | - | 0.41 | 0.17 |
| **A(24)DS(18)** | 5.75 | 12.65 | 15.40 | 53.41 | 83.24 | 116.38 | 1.23 | 1.64 | 0.71 | 1.11 |
| **A(18)DS(24)** | 110.95 | 94.40 | 98.55 | 639.32 | 981.20 | 768.39 | 0.13 | 0.25 | 0.50 | 0.30 |
| **A(16)DS(26)** | 19.34 | 15.32 | 17.81 | 203.55 | 213.72 | 166.84 | 0.10 | 0.27 | 0.00 | -0.27 |
| **A(27)DS(16)** | 3.32 | 4.17 | 3.74 | 41.86 | 37.48 | 45.30 | 0.28 | 0.02 | -0.20 | 0.09 |
| **A(25)DS(18)** | 5.22 | 6.67 | 7.32 | 30.58 | 34.61 | 46.86 | 0.58 | 0.66 | 0.24 | 0.47 |
| **A(24)DS(19)** | 8.25 | 5.14 | 5.78 | 23.07 | 23.16 | 34.63 | -0.42 | -0.46 | 0.11 | 0.55 |
| **A(23)DS(20)** | 10.64 | 9.85 | 8.95 | 30.00 | 35.40 | 34.81 | -0.06 | -0.20 | 0.23 | 0.26 |
| **A(26)DS(18)** | 6.77 | 13.55 | 15.38 | 40.51 | 44.49 | 64.54 | 1.08 | 1.31 | 0.14 | 0.69 |
| **A(24)DS(20)** | 25.42 | 29.41 | 32.70 | 92.98 | 83.41 | 113.79 | 0.34 | 0.43 | -0.19 | 0.31 |
| **A(22)DS(22)** | 4.97 | 3.83 | 4.02 | 25.06 | 21.50 | 28.39 | 0.10 | 0.11 | -0.28 | 0.09 |
| **A(26)DS(19)** | 2.51 | 3.00 | 2.81 | 12.67 | 11.93 | 11.29 | 0.50 | 0.29 | -0.04 | -0.12 |
| **A(25)DS(20)** | 5.12 | 6.48 | 8.99 | 31.71 | 33.12 | 40.96 | 0.37 | 0.86 | -0.05 | 0.40 |
| **A(24)DS(21)** | 0.00 | 0.00 | 0.00 | 0.00 | 0.00 | 0.00 | - | - | - | - |
| **A(23)DS(22)** | 3.76 | 4.06 | 4.25 | 14.11 | 13.43 | 13.77 | 0.09 | 0.08 | -0.15 | 0.07 |
| **A(26)DS(20)** | 9.88 | 15.42 | 15.13 | 41.84 | 39.19 | 42.12 | 0.75 | 0.65 | -0.04 | 0.09 |
| **A(24)DS(22)** | 6.22 | 7.60 | 5.92 | 12.62 | 11.04 | 12.45 | 0.39 | -0.14 | -0.24 | -0.01 |
| **AH** |  |  |  |  |  |  |  |  |  |  |
| **A(24)H(14)** | 0.00 | 0.00 | 0.00 | 0.00 | 0.00 | 0.00 | - | - | - | - |
| **A(22)H(16)** | 0.11 | 0.00 | 0.00 | 1.97 | 3.26 | 3.72 | - | - | - | - |
| **A(20)H(18)** | 5.46 | 6.91 | 9.83 | 12.52 | 10.30 | 19.40 | 0.36 | 0.76 | -0.37 | 0.61 |
| **A(25)H(14)** | 0.00 | 0.00 | 0.00 | 0.00 | 0.00 | 0.00 | -- | - | - | - |
| **A(24)H(15)** | 0.00 | 0.00 | 0.00 | 0.00 | 0.00 | 0.00 | - | - | - | - |
| **A(23)H(16)** | 0.00 | 0.00 | 0.00 | 0.00 | 0.00 | 0.00 | - | - | - | - |
| **A(22)H(17)** | 0.00 | 0.00 | 0.00 | 0.00 | 0.00 | 0.00 | - | - | - | - |
| **A(26)H(14)** | 0.00 | 0.00 | 0.00 | 0.00 | 1.16 | 1.65 | - | - | - | - |
| **A(24)H(16)** | 1.28 | 3.11 | 3.85 | 32.73 | 43.36 | 47.72 | 1.32 | 1.15 | 0.25 | 0.22 |
| **A(22)H(18)** | 0.89 | 1.54 | 0.81 | 5.31 | 6.25 | 6.01 | - | - | 0.14 | 0.06 |
| **A(26)H(15)** | 0.35 | 0.78 | 0.71 | 9.51 | 11.69 | 12.00 | - | - | 0.13 | 0.13 |
| **A(25)H(16)** | 1.08 | 1.29 | 3.08 | 34.61 | 50.24 | 51.62 | - | - | 0.58 | 0.51 |
| **A(24)H(17)** | 11.31 | 19.87 | 21.73 | 200.97 | 280.83 | 300.31 | 0.81 | 0.72 | 0.52 | 0.50 |
| **A(28)H(14)** | 0.66 | 0.58 | 0.81 | 9.46 | 13.06 | 14.38 | - | - | 0.61 | 0.56 |
| **A(26)H(16)** | 10.12 | 16.04 | 16.76 | 198.71 | 222.90 | 290.95 | 0.59 | 0.60 | 0.16 | 0.43 |
| **A(24)H(18)** | 37.13 | 60.71 | 62.62 | 480.04 | 637.32 | 764.19 | 0.73 | 0.70 | 0.42 | 0.57 |
| **A(28)H(15)** | 2.44 | 3.66 | 3.66 | 46.37 | 46.61 | 55.67 | 0.41 | - | 0.04 | 0.19 |
| **A(27)H(16)** | 1.74 | 2.19 | 2.82 | 27.08 | 32.82 | 39.07 | - | - | 0.35 | 0.50 |
| **A(26)H(17)** | 39.94 | 55.78 | 64.41 | 710.06 | 819.61 | 967.14 | 0.60 | 0.71 | 0.24 | 0.36 |
| **A(25)H(18)** | 48.13 | 67.34 | 73.96 | 811.54 | 963.52 | 1210.45 | 0.57 | 0.52 | 0.28 | 0.48 |
| **A(24)H(19)** | 11.87 | 16.13 | 16.77 | 194.08 | 203.22 | 284.29 | 0.63 | 0.52 | 0.08 | 0.41 |
| **A(28)H(16)** | 0.00 | 0.44 | 0.00 | 32.89 | 43.24 | 49.29 | - | - | 0.41 | 0.55 |
| **A(26)H(18)** | 50.25 | 76.46 | 90.44 | 1210.84 | 1492.34 | 1579.50 | 0.66 | 0.84 | 0.35 | 0.38 |
| **A(24)H(20)** | 76.47 | 67.30 | 80.07 | 788.01 | 897.38 | 975.71 | 0.14 | 0.27 | 0.20 | 0.32 |
| **A(28)H(17)** | 3.76 | 2.87 | 3.13 | 31.36 | 40.80 | 31.82 | - | - | 0.32 | -0.01 |
| **A(27)H(18)** | 23.80 | 18.79 | 21.32 | 190.10 | 276.63 | 205.49 | 0.09 | 0.16 | 0.40 | 0.06 |
| **A(26)H(19)** | 34.07 | 29.12 | 34.35 | 375.75 | 525.40 | 423.84 | 0.20 | 0.32 | 0.37 | 0.20 |
| **A(25)H(20)** | 34.04 | 28.48 | 33.80 | 351.21 | 490.34 | 433.85 | 0.10 | 0.17 | 0.37 | 0.28 |
| **A(28)H(18)** | 6.25 | 7.36 | 8.61 | 83.53 | 89.64 | 120.85 | 0.53 | 0.62 | 0.15 | 0.43 |
| **A(26)H(20)** | 148.49 | 133.10 | 177.10 | 1503.41 | 1628.14 | 1850.57 | 0.28 | 0.50 | 0.09 | 0.25 |
| **A(24)H(22)** | 9.78 | 7.23 | 10.00 | 58.02 | 56.26 | 67.03 | 0.08 | 0.19 | -0.01 | 0.21 |
| **A(29)H(18)** | 0.00 | 0.00 | 0.00 | 0.00 | 0.00 | 0.00 | - | - | - | - |
| **A(28)H(19)** | 7.53 | 5.31 | 6.24 | 41.17 | 41.70 | 51.86 | -0.14 | -0.15 | 0.17 | 0.24 |
| **A(27)H(20)** | 32.35 | 25.39 | 25.21 | 200.72 | 185.21 | 261.71 | 0.02 | -0.20 | 0.00 | 0.29 |
| **A(26)H(21)** | 20.09 | 17.28 | 15.06 | 111.35 | 95.41 | 165.32 | 0.24 | -0.14 | -0.09 | 0.48 |
| **A(30)H(18)** | 2.54 | 3.84 | 4.27 | 11.24 | 18.58 | 18.28 | 0.83 | 0.92 | 0.78 | 0.71 |
| **A(28)H(20)** | 37.37 | 35.09 | 38.45 | 271.34 | 235.19 | 310.23 | 0.14 | 0.03 | -0.10 | 0.19 |
| **A(26)H(22)** | 18.21 | 15.02 | 16.38 | 107.23 | 87.78 | 119.54 | 0.03 | -0.12 | -0.25 | 0.13 |
| **AP** |  |  |  |  |  |  |  |  |  |  |
| **A(22)P(16)** | 7.58 | 2.00 | 1.86 | 8.28 | 7.85 | 7.01 | - | - | 0.08 | -0.27 |
| **A(24)P(16)** | 35.13 | 29.11 | 34.35 | 415.05 | 443.24 | 622.74 | 0.12 | 0.20 | 0.13 | 0.44 |
| **A(22)P(18)** | 42.22 | 20.38 | 15.09 | 47.39 | 52.80 | 55.95 | -0.73 | -1.12 | 0.40 | 0.44 |
| **A(20)P(20)** | 1.23 | 1.25 | 1.26 | 8.60 | 13.61 | 12.97 | - | - | 0.61 | 0.42 |
| **A(24)P(17)** | 91.94 | 79.62 | 94.01 | 991.37 | 1030.95 | 1355.91 | -0.02 | 0.11 | 0.11 | 0.37 |
| **A(22)P(19)** | 9.00 | 3.96 | 3.35 | 20.35 | 19.40 | 20.34 | -0.67 | -1.20 | 0.02 | 0.02 |
| **A(26)P(16)** | 13.43 | 12.98 | 15.92 | 257.79 | 290.72 | 271.40 | 0.16 | 0.30 | 0.16 | 0.07 |
| **A(24)P(18)** | 96.79 | 91.95 | 104.83 | 999.57 | 1229.63 | 1380.86 | -0.03 | 0.17 | 0.34 | 0.45 |
| **A(22)P(20)** | 3.80 | 4.45 | 4.06 | 32.65 | 40.25 | 47.28 | - | - | 0.30 | 0.52 |
| **A(26)P(17)** | 65.15 | 50.90 | 59.92 | 657.88 | 675.63 | 596.44 | 0.00 | 0.09 | -0.02 | -0.12 |
| **A(25)P(18)** | 104.53 | 69.37 | 75.54 | 667.66 | 807.62 | 693.62 | -0.15 | -0.13 | 0.19 | 0.09 |
| **A(24)P(19)** | 54.18 | 34.10 | 33.98 | 334.19 | 389.84 | 330.57 | -0.31 | -0.33 | 0.11 | 0.03 |
| **A(26)P(18)** | 59.74 | 79.90 | 92.81 | 824.35 | 815.52 | 1013.72 | 0.55 | 0.68 | 0.05 | 0.28 |
| **A(24)P(20)** | 67.94 | 92.06 | 102.86 | 1054.54 | 940.01 | 1298.86 | 0.50 | 0.60 | -0.07 | 0.29 |
| **A(26)P(19)** | 60.15 | 54.51 | 54.86 | 468.34 | 291.49 | 448.65 | 0.07 | -0.09 | -0.41 | 0.05 |
| **A(25)P(20)** | 33.80 | 22.25 | 23.66 | 206.65 | 161.50 | 211.72 | -0.38 | -0.37 | -0.26 | 0.07 |
| **A(24)P(21)** | 65.59 | 61.99 | 64.46 | 541.94 | 384.00 | 556.19 | 0.06 | 0.03 | -0.33 | 0.11 |
| **A(26)P(20)** | 112.89 | 146.90 | 141.70 | 917.84 | 616.56 | 843.26 | 0.37 | 0.28 | -0.42 | 0.01 |
| **A(24)P(22)** | 144.46 | 166.38 | 156.98 | 1118.01 | 595.12 | 784.50 | 0.30 | 0.11 | -0.59 | -0.19 |
| **A(26)P(22)** | 46.28 | 51.78 | 44.51 | 351.57 | 173.63 | 173.40 | 0.19 | -0.06 | -0.53 | -0.50 |
| **AS** |  |  |  |  |  |  |  |  |  |  |
| **A(24)S(16)** | 50.98 | 42.99 | 51.26 | 450.08 | 532.61 | 529.27 | -0.14 | 0.03 | 0.23 | 0.17 |
| **A(25)S(16)** | 11.12 | 7.67 | 9.02 | 115.57 | 181.32 | 143.93 | -0.03 | 0.15 | 0.53 | 0.29 |
| **A(24)S(17)** | 10.89 | 15.43 | 13.80 | 128.11 | 212.93 | 183.46 | 0.44 | 0.40 | 0.67 | 0.46 |
| **A(23)S(18)** | 379.99 | 458.11 | 360.93 | 566.34 | 728.60 | 802.40 | 0.32 | 0.01 | 0.35 | 0.50 |
| **A(26)S(16)** | 46.88 | 35.00 | 50.16 | 430.54 | 541.01 | 445.82 | 0.03 | 0.49 | 0.25 | 0.03 |
| **A(24)S(18)** | 149.24 | 249.42 | 256.44 | 655.52 | 1624.96 | 1168.56 | 0.88 | 1.07 | 1.20 | 0.85 |
| **A(27)S(16)** | 3.79 | 2.09 | 1.85 | 16.15 | 15.91 | 21.11 | -0.35 | -0.65 | 0.09 | 0.15 |
| **A(26)S(17)** | 25.22 | 28.74 | 26.38 | 196.11 | 256.36 | 289.43 | 0.55 | 0.39 | 0.45 | 0.50 |
| **A(25)S(18)** | 33.21 | 50.27 | 45.71 | 174.23 | 315.56 | 331.95 | 0.64 | 0.59 | 0.86 | 0.92 |
| **A(24)S(19)** | 7.16 | 8.76 | 8.12 | 51.58 | 66.00 | 85.24 | 0.48 | 0.32 | 0.46 | 0.66 |
| **A(23)S(20)** | 1.79 | 1.91 | 1.34 | 8.61 | 8.12 | 12.25 | 0.20 | -0.28 | 0.02 | 0.26 |
| **A(22)S(21)** | 0.00 | 0.00 | 0.00 | 0.00 | 0.00 | 0.00 | - | - | - | - |
| **A(26)S(18)** | 85.40 | 206.01 | 220.89 | 731.20 | 1279.93 | 1294.38 | 1.25 | 1.52 | 0.81 | 0.84 |
| **A(24)S(20)** | 62.10 | 70.93 | 77.92 | 382.79 | 358.98 | 506.39 | 0.40 | 0.45 | 0.05 | 0.47 |
| **A(27)S(18)** | 18.77 | 9.33 | 8.31 | 32.24 | 49.34 | 38.96 | -0.94 | -1.10 | 0.89 | 0.52 |
| **A(26)S(19)** | 16.01 | 21.16 | 22.35 | 110.24 | 115.80 | 133.54 | 0.52 | 0.54 | 0.16 | 0.34 |
| **A(24)S(21)** | 7.55 | 8.24 | 7.85 | 47.64 | 28.71 | 42.41 | 0.21 | 0.03 | -0.40 | 0.04 |
| **A(23)S(22)** | 0.00 | 0.00 | 0.00 | 0.00 | 0.00 | 0.00 | - | - | - | - |
| **A(26)S(20)** | 82.83 | 138.33 | 134.76 | 719.14 | 677.07 | 625.45 | 0.84 | 0.74 | 0.07 | -0.03 |
| **A(24)S(22)** | 68.39 | 43.63 | 32.50 | 32.03 | 36.55 | 31.14 | -0.45 | -0.75 | 0.07 | - |
| **A(28)S(19)** | 1.18 | 0.49 | 1.05 | 5.30 | 7.13 | 5.12 | - | - | - | -0.09 |
| **A(25)S(22)** | 6.40 | 7.92 | 8.58 | 32.80 | 21.75 | 21.89 | 0.44 | 0.43 | -0.52 | -0.42 |
| **A(26)S(22)** | 17.62 | 27.67 | 26.14 | 243.38 | 98.42 | 95.39 | 0.78 | 0.54 | -0.77 | -0.60 |
| **NDS** |  |  |  |  |  |  |  |  |  |  |
| **N(24)DS(16)** | 61.42 | 48.44 | 46.78 | 277.09 | 398.00 | 303.20 | -0.14 | -0.22 | 0.40 | 0.19 |
| **N(22)DS(18)** | 468.53 | 381.12 | 339.06 | 718.42 | 1152.87 | 1107.15 | -0.09 | -0.23 | 0.64 | 0.68 |
| **N(20)DS(20)** | 93.76 | 37.61 | 36.38 | 126.15 | 149.70 | 134.08 | -1.02 | -1.07 | 0.08 | 0.16 |
| **N(18)DS(22)** | 352.32 | 259.59 | 245.20 | 615.33 | 988.91 | 915.83 | -0.24 | -0.29 | 0.61 | 0.63 |
| **N(16)DS(24)** | 46.20 | 32.70 | 32.92 | 215.50 | 324.48 | 249.21 | -0.23 | -0.27 | 0.42 | 0.26 |
| **N(25)DS(16)** | 11.06 | 8.35 | 10.20 | 69.54 | 96.86 | 98.44 | -0.24 | 0.00 | 0.45 | 0.55 |
| **N(24)DS(17)** | 49.16 | 41.61 | 45.29 | 186.25 | 237.56 | 273.82 | -0.04 | 0.00 | 0.29 | 0.52 |
| **N(23)DS(18)** | 122.88 | 102.28 | 104.49 | 207.88 | 306.19 | 314.64 | -0.11 | -0.12 | 0.50 | 0.61 |
| **N(22)DS(19)** | 51.80 | 25.83 | 26.53 | 59.43 | 69.80 | 67.24 | -0.88 | -0.97 | 0.12 | 0.25 |
| **N(26)DS(16)** | 50.94 | 42.47 | 50.01 | 353.53 | 362.91 | 393.48 | -0.06 | 0.09 | 0.08 | 0.22 |
| **N(24)DS(18)** | 1518.20 | 1544.37 | 1348.51 | 2514.52 | 3100.11 | 4038.65 | 0.06 | -0.05 | 0.36 | 0.72 |
| **N(20)DS(22)** | 122.97 | 57.64 | 54.44 | 154.10 | 128.91 | 171.38 | -0.77 | -1.06 | -0.09 | 0.32 |
| **N(18)DS(24)** | 954.63 | 941.74 | 856.54 | 1543.19 | 1955.10 | 2541.08 | 0.02 | -0.03 | 0.40 | 0.76 |
| **N(16)DS(26)** | 45.19 | 39.26 | 42.76 | 327.59 | 332.74 | 345.30 | 0.02 | 0.07 | 0.03 | 0.09 |
| **N(26)DS(17)** | 38.11 | 39.44 | 44.18 | 219.00 | 255.41 | 274.73 | 0.21 | 0.31 | 0.22 | 0.34 |
| **N(25)DS(18)** | 108.67 | 132.92 | 127.01 | 364.29 | 487.53 | 560.94 | 0.46 | 0.39 | 0.47 | 0.69 |
| **N(24)DS(19)** | 124.82 | 84.52 | 84.49 | 275.11 | 265.15 | 325.95 | -0.38 | -0.56 | -0.03 | 0.30 |
| **N(23)DS(20)** | 80.39 | 46.17 | 42.51 | 131.34 | 115.44 | 129.01 | -0.63 | -0.91 | -0.07 | 0.06 |
| **N(26)DS(18)** | 206.49 | 355.88 | 443.88 | 1149.71 | 1865.73 | 1701.57 | 0.90 | 1.27 | 0.79 | 0.67 |
| **N(24)DS(20)** | 404.78 | 370.54 | 350.24 | 1240.90 | 1341.80 | 1426.99 | 0.04 | -0.04 | 0.16 | 0.29 |
| **N(22)DS(22)** | 59.95 | 43.24 | 35.53 | 129.86 | 109.06 | 121.46 | -0.37 | -0.83 | -0.24 | -0.11 |
| **N(20)DS(24)** | 256.13 | 234.67 | 234.75 | 767.93 | 881.76 | 901.99 | 0.06 | 0.05 | 0.22 | 0.30 |
| **N(18)DS(26)** | 124.09 | 216.33 | 264.31 | 699.35 | 1151.34 | 1042.42 | 0.91 | 1.25 | 0.80 | 0.66 |
| **N(28)DS(17)** | 5.79 | 6.28 | 7.56 | 41.22 | 43.08 | 50.75 | 0.11 | 0.42 | 0.10 | 0.31 |
| **N(27)DS(18)** | 12.39 | 17.66 | 19.10 | 81.59 | 97.35 | 103.74 | 0.63 | 0.71 | 0.32 | 0.46 |
| **N(26)DS(19)** | 46.85 | 45.52 | 49.14 | 227.03 | 260.89 | 267.64 | 0.17 | 0.26 | 0.22 | 0.31 |
| **N(25)DS(20)** | 104.46 | 101.22 | 106.75 | 430.66 | 456.99 | 497.76 | 0.12 | 0.13 | 0.14 | 0.30 |
| **N(24)DS(21)** | 73.10 | 66.93 | 65.59 | 287.28 | 275.32 | 279.86 | 0.05 | -0.11 | 0.02 | 0.06 |
| **N(23)DS(22)** | 26.41 | 24.22 | 21.60 | 64.76 | 60.58 | 62.37 | 0.07 | -0.30 | -0.01 | 0.05 |
| **N(22)DS(23)** | 20.36 | 20.01 | 17.09 | 51.89 | 44.85 | 48.89 | 0.12 | -0.36 | -0.04 | 0.11 |
| **N(28)DS(18)** | 21.88 | 39.92 | 42.01 | 203.68 | 226.06 | 251.75 | 0.86 | 1.00 | 0.10 | 0.40 |
| **N(26)DS(20)** | 192.89 | 257.86 | 331.06 | 1143.36 | 1174.19 | 1353.03 | 0.44 | 0.83 | -0.06 | 0.29 |
| **N(24)DS(22)** | 141.26 | 180.81 | 179.16 | 549.19 | 532.57 | 543.18 | 0.42 | 0.37 | -0.12 | 0.05 |
| **N(22)DS(24)** | 101.69 | 129.91 | 120.49 | 385.68 | 355.30 | 366.18 | 0.40 | 0.24 | -0.18 | -0.01 |
| **N(20)DS(26)** | 107.68 | 143.11 | 176.68 | 644.58 | 664.85 | 761.95 | 0.46 | 0.76 | -0.03 | 0.29 |
| **N(28)DS(19)** | 5.99 | 6.77 | 7.89 | 47.73 | 48.73 | 54.43 | 0.15 | 0.40 | 0.07 | 0.24 |
| **N(27)DS(20)** | 15.25 | 22.55 | 24.26 | 116.63 | 110.02 | 124.44 | 0.57 | 0.58 | -0.19 | 0.07 |
| **N(26)DS(21)** | 30.11 | 43.91 | 51.65 | 205.27 | 197.77 | 215.54 | 0.55 | 0.68 | -0.10 | 0.14 |
| **N(25)DS(22)** | 32.64 | 48.76 | 52.56 | 153.26 | 141.45 | 142.97 | 0.59 | 0.62 | -0.23 | -0.07 |
| **N(24)DS(23)** | 15.99 | 24.97 | 24.45 | 59.88 | 50.59 | 49.23 | 0.65 | 0.56 | -0.25 | -0.26 |
| **N(23)DS(24)** | 12.90 | 22.71 | 21.32 | 54.33 | 49.11 | 45.88 | 0.85 | 0.70 | -0.16 | -0.13 |
| **N(22)DS(25)** | 23.37 | 31.96 | 34.57 | 102.40 | 87.64 | 93.26 | 0.54 | 0.56 | -0.33 | -0.07 |
| **N(28)DS(20)** | 26.48 | 48.23 | 47.34 | 205.91 | 215.01 | 237.48 | 0.81 | 0.83 | -0.15 | 0.19 |
| **N(26)DS(22)** | 52.29 | 104.23 | 98.79 | 254.95 | 276.78 | 291.22 | 0.97 | 0.90 | -0.03 | 0.19 |
| **N(24)DS(24)** | 53.51 | 113.20 | 104.78 | 196.66 | 182.50 | 203.23 | 1.07* | 0.95 | -0.24 | -0.03 |
| **N(22)DS(26)** | 32.85 | 67.12 | 62.46 | 161.30 | 153.95 | 165.37 | 0.97* | 0.92 | -0.23 | 0.03 |
| **N(29)DS(20)** | 2.46 | 4.31 | 4.09 | 18.37 | 18.83 | 22.76 | 0.85* | 0.76 | 0.01 | 0.39 |
| **N(28)DS(21)** | 4.19 | 9.36 | 7.21 | 28.55 | 25.06 | 29.43 | 1.18* | 0.79 | -0.29 | 0.08 |
| **N(27)DS(22)** | 3.02 | 9.31 | 8.48 | 20.90 | 15.22 | 16.84 | 1.25* | 1.22 | -0.45 | -0.26 |
| **N(26)DS(23)** | 5.04 | 11.01 | 8.85 | 19.82 | 15.97 | 18.81 | 1.12* | 0.77 | -0.23 | -0.01 |
| **N(25)DS(24)** | 11.78 | 26.12 | 22.79 | 41.28 | 30.16 | 41.42 | 1.17* | 0.92 | -0.47 | 0.01 |
| **N(24)DS(25)** | 11.02 | 28.63 | 21.52 | 35.83 | 31.01 | 35.53 | 1.38* | 0.96 | -0.29 | -0.13 |
| **N(23)DS(26)** | 4.73 | 11.23 | 8.68 | 14.20 | 11.61 | 15.48 | 1.26 | 0.86 | -0.33 | 0.07 |
| **N(22)DS(27)** | 3.16 | 5.78 | 5.83 | 13.94 | 10.59 | 11.38 | 0.88 | 0.79 | -0.62 | -0.22 |
| **N(30)DS(20)** | 3.73 | 7.79 | 5.75 | 23.29 | 21.86 | 25.46 | 1.00* | 0.60 | -0.28 | 0.07 |
| **N(28)DS(22)** | 7.56 | 15.46 | 13.04 | 33.51 | 25.27 | 31.17 | 1.04* | 0.76 | -0.44 | -0.16 |
| **N(26)DS(24)** | 19.12 | 42.98 | 36.50 | 57.99 | 45.30 | 52.24 | 1.20* | 0.95 | -0.34 | -0.34 |
| **N(24)DS(26)** | 20.82 | 45.40 | 37.04 | 60.14 | 42.16 | 56.79 | 1.18* | 0.88 | -0.41 | -0.24 |
| **N(30)DS(21)** | 0.39 | 0.83 | 0.86 | 1.63 | 1.56 | 0.46 | - | - | - | - |
| **N(29)DS(22)** | 38.27 | 41.26 | 35.70 | 63.04 | 56.33 | 61.80 | -0.14 | -0.21 | -0.20 | 0.04 |
| **N(28)DS(23)** | 0.00 | 0.80 | 0.08 | 0.21 | 0.80 | 0.38 | - | - | - | - |
| **N(27)DS(24)** | 0.92 | 1.48 | 1.36 | 2.71 | 0.88 | 1.98 | 0.72 | 0.59 | - | - |
| **N(26)DS(25)** | 2.50 | 7.38 | 6.08 | 8.67 | 7.27 | 6.54 | 1.62* | 1.29 | -0.41 | -1.05 |
| **N(25)DS(26)** | 3.06 | 6.64 | 4.92 | 8.39 | 5.95 | 9.15 | 1.16 | 0.69 | -0.48 | -0.53 |
| **N(24)DS(27)** | 0.30 | 1.52 | 0.72 | 0.60 | 1.04 | 1.21 | - | - | - | - |
| **N(30)DS(22)** | 9.85 | 6.24 | 2.99 | 18.33 | 9.45 | 9.43 | - | - | - | - |
| **N(28)DS(24)** | 1.95 | 0.39 | 0.00 | 1.44 | 1.30 | 0.99 | - | - | - | - |
| **N(26)DS(26)** | 0.00 | 0.00 | 0.00 | 0.00 | 0.00 | 0.00 | - | - | - | - |
| **N(29)DS(24)** | 15.58 | 12.81 | 11.00 | 26.91 | 29.30 | 28.24 | -0.43 | -0.50 | 0.12 | 0.08 |
| **N(28)DS(25)** | 0.00 | 0.00 | 0.00 | 0.00 | 0.00 | 0.00 | - | - | - | - |
| **N(27)DS(26)** | 0.00 | 0.00 | 0.00 | 0.00 | 0.00 | 0.00 | - | - | - | - |
| **N(26)DS(27)** | 0.00 | 0.00 | 0.00 | 0.00 | 0.00 | 0.00 | - | - | - | - |
| **N(30)DS(24)** | 1.99 | 0.38 | 0.17 | 1.71 | 0.96 | 0.00 | - | - | - | - |
| **N(28)DS(26)** | 0.00 | 0.00 | 0.00 | 0.00 | 0.00 | 0.00 | - | - | - | - |
| **N(26)DS(28)** | 0.00 | 0.00 | 0.00 | 0.00 | 0.00 | 0.00 | - | - | - | - |
| **NH** |  |  |  |  |  |  |  |  |  |  |
| **N(24)H(16)** | 0.73 | 0.80 | 0.65 | 4.40 | 7.04 | 7.54 | 0.08 | -0.32 | - | 0.53 |
| **N(26)H(14)** | 4.25 | 7.70 | 7.89 | 63.79 | 93.51 | 97.46 | 0.62 | 0.62 | 0.44 | 0.48 |
| **N(26)H(15)** | 0.93 | 2.82 | 3.63 | 48.91 | 60.94 | 75.37 | - | - | 0.11 | 0.35 |
| **N(25)H(16)** | 1.26 | 3.40 | 3.43 | 51.42 | 60.44 | 74.89 | - | - | 0.02 | 0.27 |
| **N(24)H(17)** | 35.64 | 60.06 | 56.60 | 413.92 | 526.03 | 645.77 | 0.80 | 0.62 | 0.30 | 0.48 |
| **N(23)H(18)** | 11.35 | 15.00 | 13.42 | 75.81 | 100.67 | 105.71 | 0.51 | 0.34 | 0.41 | 0.44 |
| **N(26)H(16)** | 42.44 | 37.73 | 48.63 | 369.97 | 412.85 | 414.27 | -0.17 | 0.43 | 0.17 | 0.17 |
| **N(24)H(18)** | 259.62 | 340.92 | 333.55 | 2976.91 | 3528.06 | 3904.82 | 0.42 | 0.45 | 0.27 | 0.38 |
| **N(26)H(17)** | 42.11 | 53.65 | 60.01 | 747.83 | 1006.54 | 891.68 | 0.37 | 0.58 | 0.36 | 0.19 |
| **N(25)H(18)** | 34.98 | 44.05 | 48.62 | 646.94 | 806.29 | 831.33 | 0.40 | 0.63 | 0.28 | 0.29 |
| **N(28)H(16)** | 24.05 | 23.36 | 26.22 | 257.97 | 334.98 | 249.74 | 0.12 | 0.30 | 0.26 | -0.02 |
| **N(26)H(18)** | 529.62 | 511.95 | 579.92 | 5502.24 | 7167.56 | 5440.30 | 0.16 | 0.39 | 0.28 | 0.00 |
| **N(24)H(20)** | 123.69 | 94.43 | 103.57 | 1203.79 | 1627.60 | 1327.19 | -0.05 | 0.10 | 0.24 | 0.15 |
| **N(28)H(17)** | 24.78 | 22.79 | 25.72 | 233.68 | 257.73 | 265.75 | 0.08 | 0.23 | 0.13 | 0.15 |
| **N(27)H(18)** | 42.54 | 41.63 | 49.54 | 507.02 | 553.88 | 630.87 | 0.26 | 0.39 | 0.11 | 0.26 |
| **N(26)H(19)** | 33.58 | 30.19 | 36.07 | 388.66 | 435.24 | 504.11 | 0.10 | 0.33 | 0.15 | 0.34 |
| **N(25)H(20)** | 35.10 | 30.57 | 38.58 | 380.69 | 444.59 | 515.31 | 0.25 | 0.46 | 0.16 | 0.37 |
| **N(28)H(18)** | 161.43 | 163.81 | 172.93 | 1580.64 | 1671.52 | 1647.27 | 0.19 | 0.23 | 0.08 | 0.06 |
| **N(26)H(20)** | 335.35 | 275.20 | 293.59 | 2650.70 | 2407.89 | 3063.37 | 0.03 | 0.08 | -0.13 | 0.19 |
| **N(28)H(19)** | 36.93 | 39.51 | 39.69 | 278.69 | 277.02 | 306.92 | 0.35 | 0.22 | 0.03 | 0.16 |
| **N(27)H(20)** | 63.70 | 62.37 | 60.41 | 445.65 | 425.88 | 519.12 | 0.29 | 0.17 | -0.07 | 0.23 |
| **N(26)H(21)** | 24.71 | 26.02 | 27.03 | 186.40 | 173.94 | 218.68 | 0.36 | 0.35 | -0.12 | 0.22 |
| **N(30)H(19)** | 22.75 | 28.75 | 28.00 | 216.51 | 245.73 | 178.74 | 0.45 | 0.29 | 0.18 | -0.27 |
| **N(28)H(20)** | 289.17 | 280.05 | 294.51 | 2407.94 | 2456.02 | 2183.02 | 0.11 | 0.15 | 0.06 | -0.10 |
| **N(26)H(22)** | 25.45 | 24.44 | 23.92 | 158.52 | 159.08 | 163.76 | 0.16 | 0.00 | -0.10 | 0.06 |
| **N(30)H(19)** | 3.82 | 4.04 | 4.96 | 29.31 | 31.75 | 27.65 | - | - | 0.15 | -0.06 |
| **N(29)H(20)** | 22.77 | 25.75 | 25.81 | 170.13 | 171.55 | 170.29 | 0.31 | 0.20 | 0.06 | 0.02 |
| **N(28)H(21)** | 19.38 | 26.19 | 25.16 | 160.60 | 156.60 | 160.15 | 0.61 | 0.34 | 0.01 | 0.06 |
| **N(30)H(20)** | 26.24 | 35.98 | 32.14 | 295.43 | 269.00 | 282.04 | 0.49 | 0.31 | -0.19 | -0.03 |
| **N(28)H(22)** | 20.45 | 29.45 | 30.73 | 168.93 | 164.32 | 164.27 | 0.53 | 0.48 | -0.11 | 0.03 |
| **NP** |  |  |  |  |  |  |  |  |  |  |
| **N(24)P(16)** | 51.55 | 41.68 | 43.26 | 445.31 | 475.75 | 565.62 | -0.04 | 0.06 | 0.13 | 0.32 |
| **N(24)P(17)** | 28.38 | 32.52 | 34.85 | 402.62 | 501.41 | 591.55 | 0.13 | 0.19 | 0.30 | 0.43 |
| **N(26)P(16)** | 74.36 | 54.00 | 73.42 | 743.51 | 837.42 | 621.41 | -0.14 | 0.29 | 0.09 | -0.22 |
| **N(24)P(18)** | 370.97 | 322.77 | 332.45 | 2135.39 | 3230.63 | 2579.62 | 0.10 | 0.20 | 0.50 | 0.30 |
| **N(26)P(17)** | 60.28 | 68.55 | 72.53 | 873.57 | 890.36 | 1012.21 | 0.40 | 0.47 | 0.00 | 0.20 |
| **N(25)P(18)** | 51.06 | 50.84 | 59.21 | 523.36 | 627.27 | 729.12 | 0.24 | 0.43 | 0.24 | 0.45 |
| **N(24)P(19)** | 50.47 | 25.91 | 32.88 | 222.70 | 246.10 | 275.35 | -0.53 | -0.30 | 0.03 | 0.26 |
| **N(23)P(20)** | 14.47 | 10.86 | 14.57 | 76.18 | 91.62 | 93.69 | -0.03 | 0.15 | 0.15 | 0.19 |
| **N(28)P(16)** | 21.97 | 20.03 | 23.45 | 217.25 | 204.66 | 209.21 | 0.15 | 0.34 | -0.06 | -0.01 |
| **N(26)P(18)** | 329.77 | 360.63 | 417.12 | 2918.00 | 2887.04 | 3184.98 | 0.38 | 0.62 | 0.01 | 0.13 |
| **N(24)P(20)** | 218.18 | 204.11 | 196.12 | 1458.11 | 1343.87 | 1698.19 | 0.17 | 0.06 | -0.12 | 0.23 |
| **N(28)P(17)** | 39.02 | 39.24 | 40.32 | 338.03 | 306.30 | 321.23 | 0.16 | 0.16 | -0.16 | -0.06 |
| **N(27)P(18)** | 33.37 | 39.15 | 37.90 | 268.79 | 250.18 | 274.74 | 0.54 | 0.48 | -0.11 | 0.05 |
| **N(26)P(19)** | 51.44 | 47.73 | 49.43 | 369.99 | 332.35 | 380.53 | 0.03 | 0.10 | -0.19 | 0.04 |
| **N(25)P(20)** | 77.64 | 88.11 | 90.16 | 608.20 | 580.56 | 662.42 | 0.29 | 0.34 | -0.11 | 0.13 |
| **N(24)P(21)** | 67.77 | 74.67 | 71.83 | 499.17 | 483.04 | 550.96 | 0.33 | 0.19 | -0.08 | 0.15 |
| **N(28)P(18)** | 95.46 | 112.10 | 112.58 | 927.31 | 926.62 | 764.60 | 0.31 | 0.28 | 0.02 | -0.24 |
| **N(26)P(20)** | 239.90 | 295.28 | 293.21 | 2000.40 | 2205.25 | 1907.67 | 0.24 | 0.35 | 0.10 | -0.11 |
| **N(24)P(22)** | 173.46 | 221.82 | 197.99 | 1090.92 | 1118.08 | 1040.57 | 0.41 | 0.27 | -0.05 | -0.05 |
| **N(26)P(21)** | 71.28 | 95.93 | 87.79 | 534.90 | 549.21 | 530.67 | 0.49 | 0.34 | -0.03 | 0.01 |
| **N(25)P(22)** | 68.23 | 97.51 | 88.75 | 430.26 | 470.31 | 423.35 | 0.63 | 0.43 | 0.08 | 0.05 |
| **N(28)P(20)** | 59.74 | 82.24 | 89.35 | 642.21 | 529.26 | 600.30 | 0.40 | 0.56 | -0.34 | -0.14 |
| **N(26)P(22)** | 163.78 | 237.43 | 250.82 | 1160.82 | 1123.28 | 1057.33 | 0.52 | 0.61 | -0.22 | -0.12 |
| **N(24)P(24)** | 22.10 | 34.85 | 33.90 | 100.15 | 109.81 | 87.56 | 0.59 | 0.58 | -0.06 | -0.17 |
| **N(28)P(21)** | 14.80 | 28.04 | 28.39 | 191.87 | 156.44 | 161.02 | 0.85 | 0.89 | -0.40 | -0.27 |
| **N(27)P(22)** | 16.03 | 32.61 | 31.46 | 143.14 | 136.25 | 125.12 | 1.01 | 0.90 | -0.23 | -0.20 |
| **N(26)P(23)** | 8.42 | 17.23 | 17.95 | 62.73 | 61.65 | 53.58 | 0.98 | 1.07 | -0.27 | -0.22 |
| **N(30)P(20)** | 7.32 | 15.12 | 13.54 | 88.46 | 85.65 | 101.08 | 0.98 | 0.99 | -0.20 | 0.09 |
| **N(28)P(22)** | 31.83 | 62.37 | 63.75 | 262.98 | 273.56 | 285.97 | 0.89 | 1.02 | -0.15 | 0.09 |
| **N(26)P(24)** | 10.88 | 25.26 | 24.04 | 52.66 | 56.71 | 50.33 | 1.12 | 1.17 | -0.21 | -0.18 |
| **N(24)P(26)** | 6.81 | 17.63 | 14.98 | 25.68 | 23.75 | 25.29 | 1.30 | 1.15 | -0.35 | -0.19 |
| **N(30)P(21)** | 0.66 | 2.17 | 1.64 | 10.70 | 10.06 | 11.57 | 1.43 | 1.35 | -0.34 | 0.06 |
| **N(29)P(22)** | 1.44 | 4.17 | 3.69 | 13.68 | 14.76 | 13.52 | 1.45 | 1.35 | -0.20 | -0.04 |
| **N(28)P(23)** | 0.34 | 1.52 | 1.10 | 3.32 | 4.21 | 4.36 | - | - | - | - |
| **N(27)P(24)** | 0.00 | 0.88 | 0.34 | 0.67 | 1.38 | 1.13 | - | - | - | - |
| **N(26)P(25)** | 0.00 | 0.70 | 0.52 | 1.19 | 1.22 | 1.17 | - | - | - | - |
| **N(30)P(22)** | 1.92 | 4.04 | 3.68 | 15.07 | 13.52 | 13.89 | 1.06 | 1.06 | -0.36 | -0.11 |
| **N(28)P(24)** | 0.32 | 1.18 | 1.16 | 2.28 | 1.52 | 2.30 | - | - | - | - |
| **N(26)P(26)** | 0.76 | 1.32 | 2.32 | 1.99 | 1.68 | 2.78 | - | 1.46 | - | - |
| **NS** |  |  |  |  |  |  |  |  |  |  |
| **N(24)S(16)** | 433.17 | 348.22 | 374.87 | 1623.12 | 2288.41 | 1782.18 | -0.16 | -0.09 | 0.38 | 0.14 |
| **N(22)S(18)** | 1380.10 | 1660.46 | 1399.85 | 2038.31 | 2964.79 | 3039.58 | 0.27 | 0.02 | 0.52 | 0.57 |
| **N(25)S(16)** | 80.98 | 64.19 | 89.36 | 709.31 | 886.25 | 735.10 | -0.05 | 0.33 | 0.25 | 0.09 |
| **N(24)S(17)** | 852.98 | 781.19 | 854.30 | 2487.41 | 3356.57 | 2498.17 | 0.07 | 0.10 | 0.38 | 0.03 |
| **N(23)S(18)** | 1719.97 | 1682.52 | 1588.91 | 1505.41 | 2290.01 | 1785.25 | 0.08 | -0.06 | 0.51 | 0.26 |
| **N(22)S(19)** | 56.03 | 41.87 | 39.49 | 85.36 | 140.46 | 100.03 | -0.26 | -0.40 | 0.57 | 0.30 |
| **N(21)S(20)** | 2.91 | 2.69 | 2.53 | 18.85 | 30.77 | 19.66 | -0.10 | -0.14 | 0.32 | 0.07 |
| **N(26)S(16)** | 189.48 | 191.07 | 203.20 | 2046.62 | 2171.78 | 2210.48 | 0.30 | 0.40 | 0.09 | 0.14 |
| **N(24)S(18)** | 8500.66 | 9820.31 | 8029.27 | 12816.04 | 17005.76 | 18499.81 | 0.26 | -0.01 | 0.40 | 0.54 |
| **N(22)S(20)** | 25.25 | 32.69 | 32.55 | 163.51 | 203.59 | 246.57 | 0.42 | 0.38 | 0.39 | 0.60 |
| **N(20)S(22)** | 1.64 | 1.04 | 1.10 | 13.26 | 12.63 | 17.40 | -0.71 | -0.78 | -0.07 | 0.26 |
| **N(27)S(16)** | 18.45 | 19.76 | 20.23 | 219.79 | 243.34 | 197.16 | 0.18 | 0.23 | 0.13 | -0.12 |
| **N(26)S(17)** | 183.24 | 276.30 | 278.56 | 2194.20 | 2795.99 | 2193.28 | 0.62 | 0.78 | 0.35 | 0.05 |
| **N(24)S(19)** | 236.32 | 200.11 | 201.83 | 610.08 | 684.12 | 717.52 | -0.14 | -0.21 | 0.20 | 0.28 |
| **N(23)S(20)** | 43.73 | 43.84 | 40.10 | 191.09 | 202.47 | 224.73 | 0.08 | -0.11 | 0.22 | 0.28 |
| **N(22)S(21)** | 5.46 | 4.97 | 5.61 | 36.30 | 32.41 | 44.64 | -0.01 | -0.03 | 0.03 | 0.28 |
| **N(28)S(16)** | 30.02 | 49.41 | 38.03 | 407.06 | 535.45 | 311.67 | 0.71 | 0.52 | 0.38 | -0.38 |
| **N(26)S(18)** | 1151.93 | 2681.53 | 2778.06 | 9480.33 | 16185.14 | 14063.54 | 1.14 | 1.33 | 0.73 | 0.56 |
| **N(24)S(20)** | 394.08 | 581.11 | 546.98 | 1967.78 | 2166.71 | 2456.62 | 0.64 | 0.53 | 0.17 | 0.36 |
| **N(28)S(17)** | 29.26 | 50.49 | 39.90 | 414.26 | 565.91 | 354.44 | 0.71 | 0.51 | 0.45 | -0.23 |
| **N(27)S(18)** | 96.40 | 212.74 | 176.44 | 883.30 | 1549.32 | 987.60 | 1.05 | 0.89 | 0.80 | 0.19 |
| **N(26)S(19)** | 104.88 | 158.75 | 164.75 | 959.41 | 1340.49 | 1064.28 | 0.67 | 0.72 | 0.55 | 0.21 |
| **N(25)S(20)** | 123.60 | 201.96 | 194.99 | 992.05 | 1193.46 | 1051.30 | 0.78 | 0.69 | 0.37 | 0.15 |
| **N(24)S(21)** | 40.61 | 57.36 | 53.92 | 319.64 | 306.90 | 332.90 | 0.64 | 0.39 | 0.11 | 0.12 |
| **N(23)S(22)** | 7.77 | 10.33 | 9.47 | 47.05 | 44.59 | 46.37 | 0.55 | 0.27 | 0.12 | 0.04 |
| **N(28)S(18)** | 152.00 | 412.02 | 330.42 | 2276.60 | 3654.07 | 2160.17 | 1.08 | 1.15 | 0.60 | -0.11 |
| **N(26)S(20)** | 397.37 | 774.57 | 879.57 | 3588.98 | 4863.51 | 4005.53 | 0.87 | 1.17 | 0.41 | 0.18 |
| **N(24)S(22)** | 79.17 | 110.68 | 106.99 | 425.18 | 383.67 | 426.91 | 0.52 | 0.41 | -0.12 | 0.03 |
| **N(22)S(24)** | 4.14 | 6.98 | 5.27 | 13.33 | 11.86 | 14.31 | 0.75 | 0.31 | -0.23 | 0.04 |
| **N(29)S(18)** | 36.98 | 70.99 | 55.49 | 232.00 | 314.64 | 230.39 | 0.90 | 0.56 | 0.36 | -0.10 |
| **N(28)S(19)** | 20.44 | 41.84 | 41.27 | 364.92 | 419.36 | 390.49 | 0.96 | 0.94 | 0.22 | 0.01 |
| **N(27)S(20)** | 47.31 | 93.66 | 90.45 | 652.08 | 694.29 | 751.87 | 0.96 | 0.89 | 0.12 | 0.18 |
| **N(26)S(21)** | 43.72 | 78.67 | 81.62 | 500.64 | 474.13 | 602.91 | 0.84 | 0.81 | -0.02 | 0.32 |
| **N(25)S(22)** | 27.57 | 44.73 | 43.90 | 204.80 | 184.57 | 242.92 | 0.72 | 0.58 | -0.18 | 0.30 |
| **N(24)S(23)** | 5.07 | 7.81 | 6.47 | 24.07 | 20.86 | 28.85 | 0.64 | 0.20 | -0.37 | 0.35 |
| **N(23)S(24)** | 1.70 | 3.71 | 2.64 | 7.27 | 6.72 | 9.30 | 1.06 | 0.55 | -0.19 | 0.19 |
| **N(28)S(20)** | 127.35 | 275.15 | 264.57 | 1880.72 | 2059.88 | 2170.41 | 1.04 | 1.06 | 0.10 | 0.20 |
| **N(26)S(22)** | 75.10 | 159.42 | 167.01 | 706.78 | 676.64 | 868.99 | 1.03 | 1.13 | -0.13 | 0.32 |
| **N(24)S(24)** | 10.52 | 21.16 | 21.65 | 43.02 | 43.10 | 57.16 | 0.98 | 1.03 | -0.12 | 0.40 |
| **N(29)S(20)** | 11.07 | 23.65 | 22.17 | 158.75 | 158.96 | 198.43 | 1.03 | 0.93 | 0.07 | 0.38 |
| **N(28)S(21)** | 19.34 | 41.69 | 39.22 | 222.07 | 208.01 | 305.79 | 1.11 | 0.89 | -0.01 | 0.61 |
| **N(27)S(22)** | 12.37 | 25.05 | 23.46 | 92.41 | 85.50 | 128.55 | 1.01 | 0.81 | -0.02 | 0.56 |
| **N(26)S(23)** | 4.01 | 7.99 | 6.85 | 19.43 | 21.07 | 28.13 | 0.97 | 0.72 | 0.22 | 0.62 |
| **N(25)S(24)** | 3.26 | 6.80 | 5.96 | 11.43 | 9.62 | 16.75 | 1.03* | 0.84 | -0.23 | 0.56 |
| **N(24)S(25)** | 2.19 | 5.42 | 4.66 | 4.67 | 4.66 | 10.24 | 1.39 | 1.17 | -0.15 | 1.02 |
| **N(30)S(20)** | 17.58 | 35.23 | 31.45 | 222.55 | 222.80 | 245.53 | 0.93* | 0.78 | 0.02 | 0.21 |
| **N(28)S(22)** | 42.62 | 90.31 | 87.77 | 342.30 | 307.84 | 435.08 | 1.07 | 0.90 | -0.11 | 0.49 |
| **N(26)S(24)** | 8.73 | 20.34 | 17.91 | 30.93 | 27.73 | 48.32 | 1.21* | 1.01 | -0.10 | 0.53 |
| **N(24)S(26)** | 4.99 | 11.41 | 10.00 | 9.33 | 7.17 | 13.08 | 1.23* | 1.08 | -0.29 | 0.42 |
| **N(30)S(21)** | 3.45 | 8.41 | 8.23 | 34.18 | 31.10 | 45.01 | 1.22* | 1.11 | -0.14 | 0.50 |
| **N(29)S(22)** | 7.29 | 15.50 | 15.30 | 51.42 | 38.97 | 60.36 | 1.07 | 0.87 | -0.31 | 0.36 |
| **N(28)S(23)** | 1.88 | 4.37 | 4.10 | 7.02 | 6.95 | 12.98 | 1.22* | 1.01 | 0.04 | 0.85 |
| **N(26)S(25)** | 1.51 | 4.17 | 3.66 | 4.34 | 3.30 | 6.02 | 1.47* | 1.27 | -0.08 | 0.60 |
| **N(25)S(26)** | 1.44 | 3.07 | 2.64 | 1.91 | 1.45 | 3.40 | 1.24* | 0.96 | -0.70 | -0.11 |
| **N(30)S(22)** | 11.29 | 24.19 | 22.86 | 71.84 | 61.19 | 95.51 | 1.02 | 0.80 | -0.10 | 0.53 |
| **N(28)S(24)** | 1.95 | 5.46 | 4.78 | 6.18 | 4.89 | 11.96 | 1.49* | 1.12 | -0.15 | 0.76 |
| **N(26)S(26)** | 3.69 | 6.88 | 6.10 | 5.64 | 4.24 | 12.55 | 1.01 | 0.80 | -0.34 | 0.86 |
| **EOH** |  |  |  |  |  |  |  |  |  |  |
| **E(18:2)O(30)H(18)** | 0.00 | 0.00 | 0.00 | 831.79 | 799.85 | 852.20 | - | - | 0.35 | 0.48 |
| **E(18:2)O(28)H(20)** | 0.00 | 0.00 | 0.00 | 54.33 | 42.27 | 47.00 | - | - | 0.20 | 0.40 |
| **E(18:2)O(31)H(18)** | 0.00 | 0.00 | 0.00 | 128.40 | 109.33 | 116.78 | - | - | 0.20 | 0.31 |
| **E(18:2)O(30)H(19)** | 0.00 | 0.00 | 0.00 | 141.53 | 112.83 | 136.70 | - | - | 0.01 | 0.31 |
| **E(18:2)O(29)H(20)** | 0.00 | 0.00 | 0.00 | 65.99 | 51.54 | 61.83 | - | - | 0.17 | 0.52 |
| **E(18:2)O(32)H(18)** | 0.00 | 0.00 | 0.00 | 404.52 | 290.88 | 300.62 | - | - | 0.28 | 0.41 |
| **E(18:2)O(30)H(20)** | 0.00 | 0.00 | 0.00 | 627.29 | 417.04 | 468.97 | - | - | 0.04 | 0.33 |
| **E(18:2)O(32)H(19)** | 0.00 | 0.00 | 0.00 | 60.20 | 41.65 | 41.33 | - | - | 0.45 | 0.47 |
| **E(18:2)O(31)H(20)** | 0.00 | 0.00 | 0.00 | 145.83 | 86.61 | 93.36 | - | - | 0.21 | 0.41 |
| **E(18:2)O(30)H(21)** | 0.00 | 0.00 | 0.00 | 57.32 | 34.21 | 36.47 | - | - | 0.35 | 0.57 |
| **E(18:2)O(32)H(20)** | 0.00 | 0.00 | 0.00 | 334.84 | 158.66 | 189.21 | - | - | -0.02 | 0.34 |
| **E(18:2)O(30)H(22)** | 0.00 | 0.00 | 0.00 | 59.59 | 24.10 | 23.59 | - | - | 0.18 | 0.45 |
| **EOP** |  |  |  |  |  |  |  |  |  |  |
| **E(18:2)O(32)P(18)** | 0.00 | 0.00 | 0.00 | 105.14 | 52.54 | 57.66 | - | - | -0.05 | 0.17 |
| **E(18:2)O(30)P(20)** | 0.00 | 0.00 | 0.00 | 173.73 | 65.44 | 90.41 | - | - | -0.27 | 0.22 |
| **E(18:2)O(28)P(22)** | 0.00 | 0.00 | 0.00 | 12.13 | 1.91 | 2.63 | - | - | - | - |
| **E(18:2)O(31)P(20)** | 0.00 | 0.00 | 0.00 | 36.79 | 11.79 | 13.38 | - | - | 0.28 | 0.51 |
| **E(18:2)O(30)P(21)** | 0.00 | 0.00 | 0.00 | 55.16 | 13.95 | 15.16 | - | - | 0.05 | 0.39 |
| **E(18:2)O(29)P(22)** | 0.00 | 0.00 | 0.00 | 1.10 | 1.39 | 0.67 | - | - | - | - |
| **E(18:2)O(32)P(20)** | 0.00 | 0.00 | 0.00 | 79.18 | 18.94 | 23.82 | - | - | -0.12 | 0.29 |
| **E(18:2)O(30)P(22)** | 0.00 | 0.00 | 0.00 | 128.58 | 22.12 | 22.14 | - | - | 0.13 | 0.29 |
| **EOS** |  |  |  |  |  |  |  |  |  |  |
| **E(18:2)O(30)S(18)** | 0.00 | 0.00 | 0.00 | 2686.56 | 2153.34 | 2013.99 | - | - | 0.37 | 0.42 |
| **E(18:2)O(30)S(19)** | 0.00 | 0.00 | 0.00 | 593.17 | 415.20 | 470.63 | - | - | 0.21 | 0.53 |
| **E(18:2)O(29)S(20)** | 0.00 | 0.00 | 0.00 | 369.73 | 203.16 | 231.12 | - | - | 0.21 | 0.59 |
| **E(18:2)O(30)S(20)** | 0.00 | 0.00 | 0.00 | 4422.87 | 2061.04 | 2221.08 | - | - | 0.24 | 0.55 |
| **E(18:2)O(28)S(22)** | 0.00 | 0.00 | 0.00 | 171.91 | 42.49 | 39.53 | - | - | 0.34 | 0.54 |
| **E(18:2)O(32)S(19)** | 0.00 | 0.00 | 0.00 | 257.34 | 147.19 | 149.08 | - | - | 0.46 | 0.62 |
| **E(18:2)O(31)S(20)** | 0.00 | 0.00 | 0.00 | 804.64 | 352.70 | 370.60 | - | - | 0.50 | 0.83 |
| **E(18:2)O(30)S(21)** | 0.00 | 0.00 | 0.00 | 762.62 | 252.91 | 249.16 | - | - | 0.60 | 0.80 |
| **E(18:2)O(32)S(20)** | 0.00 | 0.00 | 0.00 | 1768.46 | 674.42 | 668.78 | - | - | 0.34 | 0.56 |
| **E(18:2)O(30)S(22)** | 0.00 | 0.00 | 0.00 | 2091.58 | 373.46 | 321.73 | - | - | 0.60 | 0.70 |

- Represents missing value, due to zero values in one of the treatment groups so unable to calculate log2 fold change

*P<0.05 log2FC vs control
