# Supplementary material for: Lipid functions in skin: Differential effects of n-3 polyunsaturated fatty acids on cutaneous ceramides, in a human skin organ culture model
Source: Biochim Biophys Acta. 2017 Sep;1859(9Part B):1679–89. doi: 10.1016/j.bbamem.2017.03.016 (PMC5504780; doi:10.1016/j.bbamem.2017.03.016)
Supplement: Supplementary Fig. S3 — Representative data showing the viability and metabolic competence of skin from an individual donor in ex vivo culture. Following an initial release of lactate dehydrogenase (LDH) into the culture medium following the trauma experienced by peripheral skin cells during biopsy sampling, LDH release settles within a day and remains low during culture (data are expressed as μUnits of LDH activity per ml culture medium). Neither EPA nor DHA affected viability and therefore LDH release (A). Skin biopsies cultured for 3 and 6 days with 50 μM EPA (green squares) or DHA (orange diamonds) show uptake of the fatty acids and alterations in the EPA and DHA content of the epidermis (B) and dermis (C), compared with vehicle (DMSO) treatment. EPA and DHA content are shown as % of total fatty acids in the epidermis (B) or dermis (C). [file mmc3.docx]

**Supplementary Data S3**

**Supplementary Figure S3**. Representative data showing the viability and metabolic competence of skin from an individual donor in *ex vivo* culture. Following an initial release of lactate dehydrogenase (LDH) into the culture medium following the trauma experienced by peripheral skin cells during biopsy sampling, LDH release settles within a day and remains low during culture (data are expressed as µUnits of LDH activity per ml culture medium). Neither EPA nor DHA affected viability and therefore LDH release (**A**). Skin biopsies cultured for 3 and 6 days with 50 µM EPA (green squares) or DHA (orange diamonds) show uptake of the fatty acids and alterations in the EPA and DHA content of the epidermis (**B**) and dermis (**C**), compared with vehicle (DMSO) treatment. EPA and DHA content are shown as % of total fatty acids in the epidermis (**B**) or dermis (**C**).
